# Toughening Brittle Poly(ethylene Furanoate) with Linear Low-Density Polyethylene via Interface Modulation Using Reactive Compatibilizers

**DOI:** 10.1021/acsomega.4c09301

**Published:** 2025-02-04

**Authors:** Safa Ahmed, Ruth Cardinaels, Basim Abu-Jdayil, Abdul Munam, Muhammad Z. Iqbal

**Affiliations:** 1 Chemical and Petroleum Engineering, United Arab Emirates University (UAEU), PO Box 15551, Al Ain, UAE; 2 Department of Chemical Engineering, KU Leuven, Celestijnenlaan 200J, Box 2424, Leuven, 3000 Flanders ,Belgium; 3 Department of Mechanical Engineering, Eindhoven University of Technology, P.O. Box 513, Eindhoven, MB 5600, The Netherlands; 4 Department of Biomedical Sciences, University of Niagara Falls, L2E 7J7 Ontario,Canada

## Abstract

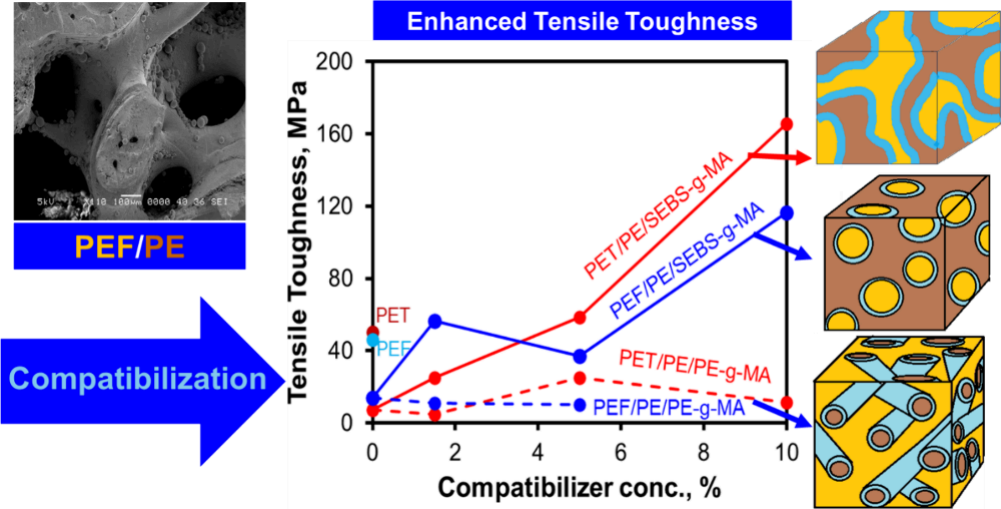

Among various biorenewable polymers, poly(2,5-ethylene furandicarboxylate) (PEF) has a large potential to replace fossil-based poly(ethylene terephthalate) (PET) for different applications. However, despite showing better gas barrier properties compared to PET, the inferior mechanical properties of PEF hinder its potential applications. This study reports the toughening of PEF with linear low-density polyethylene (PE) via melt blending by reactive compatibilization at the polymer–polymer interface and benchmarking against similar PET/PE blends. The wettability and spreading coefficient predictions indicate a preferable location of the ternary component (styrene-ethylene/butylene-styrene-graft-maleic anhydride (SEBS-*g*-MA) or polyethylene-graft-maleic anhydride (PE-*g*-MA)) along the PEF/PE interface. The interfacial ternary component (concentration and type) exhibited substantial effects on the PEF/PE morphology, altering it from a very coarse incompatible structure to a dispersed morphology for SEBS-*g*-MA, and fibrillar and cocontinuous morphologies for PE-*g*-MA. The morphology change in the blends is attributed to reactive compatibilization between the anhydride group of the compatibilizer and the hydroxyl end-group in PEF at the interface. The SEBS-*g*-MA compatibilized blends exhibited enhanced ductility, as the elongation at break substantially increased with increasing compatibilizer loading, resulting in an 800% increment in the elongation at break and 250% in the tensile toughness compared to those of the neat PEF. These improvements may open new applications of biobased PEF flexible materials for the packaging industry.

## 1 Introduction

Advancing the current state of knowledge toward biorenewable materials and their applications is currently the focus in research and industry. Biomass-derived polymers have attracted increased attention globally for manufacturing sustainable polymers to replace many existing fossil-based polymers. In this context, biorenewable poly(ethylene furandicarboxylate) (PEF) has been projected to replace the commonly used poly(ethylene terephthalate) (PET) for beverage bottles and many other applications.^1^

**Scheme 1:**
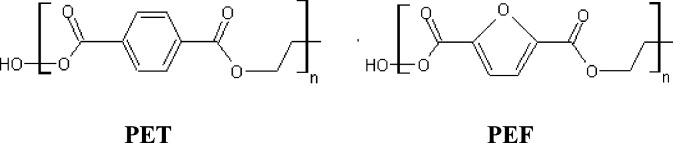
Chemical Structures of PET and PEF Repeat Units

Poly(ethylene terephthalate) (PET) is one of the polymers being produced and recycled in large quantities at the moment, especially in the packaging industry.^2,3^ However, increasing concerns over the use of fossil-based PET have led to a search for biobased alternatives of PET. Recently reported biorenewable poly(ethylene 2,5-furandicarboxylate) (PEF), the most spotlighted member of the furan-based polyester family,^1,2^ has shown promising results as an alternative for PET.^4^ The PEF contains a five-atom ring (furan) of carbon atoms, while PET contains a six-atom ring (benzene) (Scheme 1). Compared to the benzene ring, the furan ring is less aromatic due to its π-excessive character which leads to a different chemical reactivity. In addition, the geometry of the aromatic ring in furan-2,5-dicarboxylic acid (FDCA) and terephthalic acid (TA) has noticeable impacts on the rate of crystallization and degree of crystallinity.^5^

The synthesized PEF has significantly improved physical properties, justifying its attractiveness as a replacement for PET. For example, amorphous PEF exhibited an 11 times reduction in oxygen permeability compared to PET as reported by Burgess et al.^6^ The large reduction in permeability is attributed to the furan ring polarity and nonsymmetrical axis of ring rotation, which hinders complete ring flipping for PEF in addition to the significant differences in chain mobility between PEF and PET. The latter also leads to a 19 times reduction in carbon dioxide permeability and a 2.8 times reduction in water permeability in PEF compared to PET.^4,7^ On the other hand, the thermal properties of PEF were found to be close to that of PET, as both polymers show a high melting point above 210 °C, while the reported glass transition of PEF is slightly higher than that of PET. Hence, these promising properties justify PEF as a viable substitute for PET in packaging applications.

Despite its higher elastic modulus, PEF exhibits lower toughness and higher brittleness compared to PET,^8^ hindering its application as a viable replacement for PET. The high rigidity of PEF, which is associated with poor stretchability is again attributed to the suppression of furan ring-flipping due to the nonlinearity of ring rotation and ring polarity. This leads to a reduction in β-relaxation motion in PEF. For PET, both phenyl ring-flipping and carbonyl motions contribute to its relaxation behavior, which decreases its rigidity.^9^

Currently, PEF production is accelerating; e.g., Avantium, the pioneer in PEF production, is targeting the commercial production of PEF in 2024 based on a projected production of 50 kilotons per year of the monomer 2,5-Furandicarboxylic acid (FDCA). Moreover, PEFerenece, a European consortium involving spectra of 13 companies representing various industries, aims at producing PEF to use as a substitute for PET for different applications. Thus, it is necessary for academia and industry to investigate the potential of PEF and to find alternative routes to widen its spectrum of utilization.

Polymer blending is an excellent route for the optimization of properties that are barely achievable in single polymers. Owing to the low entropy of mixing, polymers are generally immiscible.^10^ This is advantageous since immiscible blends can show superior properties, which exceed that of either of their components compared to the averaged properties that might be obtained in miscible blends. In general, different morphologies can be developed in immiscible blends depending on the interfacial tension between the two polymer phases, the blend composition, the compatibilization effect, etc. Promoting and preserving nonspherical domains in the melt state are key challenges in manipulating blend morphology.^11^ A wide range of properties could be enhanced based on nonspherical microstructures. For instance, the barrier properties are significantly enhanced in lamellar morphologies, the fibrillar morphology is linked to improved processability and low thermal expansion coefficients, and cocontinuous morphologies significantly increase the electrical conductivity in the presence of conductive fillers.^12^

There are only a few reports on blending PEF with other polymers for tailored physicochemical properties. Poulopoulou et al.^13^ reported dynamic miscibility and homogeneity of PEF with miscible poly(propylene furanoate) PPF and poly(butylene furanoate) (PBF). PEF/PPF blends exhibited a single glass transition temperature (*T*_g_), whereas PEF/PBF showed two distinct *T*_g_’s, indicating immiscibility, which further merged into a single *T*_g_ during prolonged reactive compatibilization. In both cases, solution processing was utilized for manufacturing the blends. In addition, the same group later analyzed the crystallization kinetics of PEF/PBF blends and reported a decrease in the degree of crystallinity of both polymers in the blends compared to that of pure polymers for all compositions.^14^ An interesting compatibility study on PEF/PET blends^15^ revealed, based on thermal behavior, that the miscibility of PET/PEF blends depends substantially on the blend composition. At low and high fractions of PET, the blends were found to be miscible, and medium compositions resulted in partial miscibility. This finding is in line with a study that investigated the blending of PEF with postconsumer glycol modified PET (PET-G),^16^ as it was found that the PET-G/PEF 80/20 blend showed largely improved properties among the different compositions. This was attributed to interactions between PEF and PET-G polymer chains and possible reactions between their functional groups. Yang et al.^17^ improved the toughness of PEF by reactive compatibilization with polyamide 11 using a reactive chain extender. The resultant blends exhibited a significant increase in elongation at break (from 3.6 to 90.1%) and unnotched impact strength (from 3.8 to 30.3 kJ/m^2^). Fredi et al.^18^ used a reactive chain extender (Joncryl ADR) and reported fully biorenewable blends of PEF with poly(lactic acid) (PLA) via reactive blending for improving the UV-resistance and oxygen barrier properties of PLA for packaging applications. Even 1 wt % PEF in PLA improved the UV-resistance and oxygen barrier properties significantly while keeping the blends transparent. Another interesting application of PLA/PEF blends was investigated by applying a wet-spinning and drawing process to synthesize fiber blends for the textile industry.^19^ Very recently, Wang et al.^20^ incorporated PEF into poly(butylene adipate-*co*-terephthalate) (PBAT) and produced high strength and gas barrier transparent films for food packaging. The developed films showed a marked reduction in oxygen transmission as well as water vapor transmission rate through the films up to 20 wt % PEF in PBAT. Earlier, Zhang et al.^21^ blended PEF with PBAT up to 50 wt % via melt compounding. Up to 7 wt % PEF content, the PEF/PBAT blend exhibited a remarkable elongation at break (>1000%) attributed to the reduced crystallinity of PBAT due to the addition of PEF. Increasing the concentration of PEF in the blend further helped in improving the impact strength.

In this research, immiscible blends of PEF with PE aiming at enhancing the ductility of PEF are developed with the addition of polyethylene-graft-maleic anhydride (PE-*g*-MA) and styrene-ethylene/butylene-styrene-graft-maleic anhydride (SEBS-*g*-MA) as compatibilizers at different concentrations. These compatibilizers are widely used in polymer blends due to their chemical compatibility and effectiveness in improving the blend performance.^22^

The maleic anhydride (MA) groups in the PE-*g*-MA can react with the terminal group in the polyester, enhancing the interfacial adhesion between the polyester and PE phases. This reaction reduces phase separation and improves the mechanical properties. Similarly, SEBS-*g*-MA also contains MA groups that can interact with polyester, while its styrene-ethylene/butylene-styrene backbone provides compatibility with PE via diffusion. This dual compatibility facilitates a better dispersion of polyester within the PE matrix and enhances the overall blend morphology. In addition, both PE-*g*-MA and SEBS-*g*-MA have been widely reported in the literature to improve the mechanical properties of polymer blends by enhancing interfacial adhesion and promoting compatibility in immiscible polymer blends.^23−25^ Moreover, both PE-*g*-MA and SEBS-*g*-MA are readily available and cost-effective, making the development of these compatible blends economically feasible.

To the best of our knowledge, this is the first comprehensive report on PEF blends with polyolefins using SEBS-*g*-MA and PE-*g*-MA. This represents a significant advancement in the field, as previous research has not explored the compatibilization of PEF/PE blends. The use of these compatibilizers provides a fundamental understanding of the interfacial interactions and morphological effects in such blends, establishing this work as a pioneering contribution to the field of polymer blend compatibilization. Owing to the scarcity of published literature on PEF blends, a comparison is developed with similar PET/PE blends. The blends manufactured by melt blending were characterized for morphology and thermal and mechanical properties.

## 2 Experimental Section

### 2.1 Materials

Poly(ethylene 2,5-furandicarboxylate) (PEF) was purchased from Avantium, Switzerland with an intrinsic viscosity of >0.5 g/mL and a molecular weight of >30,000 g/mol. Poly(ethylene terephthalate) (PET) (BC 212) was supplied by SABIC, KSA. Linear low-density polyethylene with a density of 0.923 g/mL and a melt flow rate (190 °C/2.16 kg) of 0.25 g/10 min was purchased from Borouge, UAE. Styrene-ethylene/butylene-styrene-graft-maleic anhydride (SEBS-*g*-MA) with a solution viscosity of 1000 mPa s (25 wt % in toluene) and specific gravity of 0.91 was provided by Kraton, USA. It is a triblock copolymer based on styrene and ethylene/butylene with 30% styrene and a bound maleic anhydride content of 1.4–2.0 wt % (KRATON FG 1901G). Polyethylene-graft-maleic anhydride (PE-*g*-MA) with a viscosity of 500 cP at 140 °C was obtained from Aldrich, USA. Thermal stabilizer (Irganox 1010) was provided by BASF, Germany.

### 2.2 Preparation of Blends

Polymer blending was carried out using a corotating screw microcompounder (Minilab II, Haake, Germany) under a nitrogen atmosphere. Prior to melt processing, all materials were dried overnight under vacuum at 120 °C for PET and PEF, and at 60 °C for PE and the compatibilizers. Blending was performed by feeding all blend components simultaneously into the melt extruder with 0.1% thermal stabilizer at 50 rpm for 5 min. PEF/PE blends were processed at 240 °C and PET/PE blends were melt mixed at 270 °C. In each experiment, 2.4 g of each polymer was used and compatibilizer concentration was changed as 1.5, 5, and 10 wt %, corresponding to 0.07, 0.25, and 0.53 g, respectively. The detailed amounts are exhibited in Table 1.

**Table 1:** Amounts of Materials Used to Develop the Blends

	amount at different conc. of compatibilizer (g)			
	0 wt %	1.5 wt %	5 wt %	10 wt %
PEF	2.4	2.4	2.4	2.4
PE	2.4	2.4	2.4	2.4
compatibilizer	0	0.07	0.25	0.53

Extrudates were quenched immediately in an ice–water bath to stabilize the morphology, and thereafter, dried overnight under vacuum at 70 °C followed by pelletization. For mechanical testing and dynamic mechanical analysis (DMA), samples were molded into a rectangular shape by placing a small amount of pelletized sample into a vacuum molding insert (MeltPrep VCM, Austria), heated under vacuum for 5–8 min at 245 and 280 °C for PEF and PET blends, respectively, followed by cooling under vacuum down to room temperature at approximately 17 °C/min. Samples are denoted as PEF(PET)/PE/compatibilizer-x% and a complete sample coding and composition are listed in Table S1.

### 2.3 Blends Characterization

Morphology was examined using scanning electron microscopy (SEM, JSM-5600). Prior to SEM, PET and PEF were favorably extracted from cross-sectionally cryo-fractured extrudates after molding by soaking them in hexafluoro isopropanol (HFIP) at 60 °C for 6 days followed by drying in a vacuum oven at 60 °C for 24 h to remove solvent traces. SEM scanning was performed on the cryo-fractured surfaces after being sputter coated with gold using an autocoater at 7 Pa and 30 mA for 150 s.

The molecular weight was examined using an Agilent 1260 HPLC, USA. For analysis, a weighed amount of the sample was dissolved in an appropriate volume of HFIP and sodium trifluoroacetate (NaTFA) while shaking to obtain a concentration of 2 mg/mL. Then, 1 mL of dissolved sample was promptly transferred into HPLC vials to perform the test.

Functional groups in the polymer samples were studied using FTIR-ATR (JASCO, FT/IR-470, USA). Thin films were examined over the spectral range of 500–4000 cm^–1^, with 32 scans at 4 cm^–1^ resolution.

X-ray diffraction (XRD, Shimadzu-6100, Japan) was performed at 40 kV voltage and 30 mA current using Cu–Kα radiation (λ = 1.542 Å). The XRD scans were conducted between 5° ≤ 2θ ≤ 50° at a 2°/min scan rate at room temperature. Blends’ crystallinity was quantified by calculating the percentage ratio of the area under the crystalline peaks to the total area encompassing both crystalline and amorphous peaks in the XRD curves.

Thermal properties such as melting, crystallization, and glass transition temperatures were evaluated using a Discovery differential scanning calorimeter (DSC 25, TA Instruments, USA) under a nitrogen atmosphere (50 mL/min). Typically, 5–7 mg of sample was hermetically sealed in an aluminum pan and heated from 25 °C to 250 and 270 °C for PEF and PET blends, respectively, at 10 °C/min. Samples were held at the maximum temperature for two min to erase the thermal history followed by subsequent cooling to 25 °C at the same rate to record the crystallization transitions. A second heating cycle was performed at the same rate to report melting (*T*_m_), cold crystallization (*T*_cc_), and glass transition (*T*_g_) temperatures. *T*_g_ was taken as the midpoint of the transition curve in the second heating scan for the PEF/PE blends. Whereas, in the heating profile of PET/PE systems, the *T*_g_ of PET could not be recorded due to the mixed endothermic melting of PE starting close to the expected *T*_g_ of PET. However, the change in the thermogram baseline in the cooling profile was adequate to analyze the glass-forming temperature of the PET phase in PET/PE blends (Figure 6).

Dynamic mechanical analysis (DMA) was conducted using a DMA 242 E Artemis (Netzsch, Germany) in tension mode in the temperature ranges of 30–150 and 30–180 °C for PEF and PET blends, respectively, at a fixed frequency of 10 Hz and heating rate of 3 °C/min, applying an amplitude of 20 μm. Tested samples with dimensions of 40 × 5 × 0.5 mm were cut from molded rectangles.

Tensile testing was performed at room temperature by using a universal tensile testing machine (Shimadzu, AGS-X Series, Japan). Dog-bone test specimens with a 30 mm total length, 2 mm gauge width, and 0.25 ± 0.03 mm thickness were cut from molded rectangles and tested at an overhead speed of 2 mm/min except for PE samples, which were tested at an overhead speed of 5 mm/min. The overhead speed varies based on the anticipated mechanical properties of the tested material, particularly its elongation at break, according to the standard test method of ASTM D882–18. A minimum of three replicates were tested for each sample, and averaged results of tensile strength, elongation at break, Young’s modulus, and toughness properties were reported.

## 3 Results and Discussion

### 3.1 Prediction of Polymer Miscibility and Localization of the Compatibilizer

Polymers are generally immiscible due to low mixing entropy, and this immiscibility can enhance the properties of polymer blends. The solubility parameter (δ) is a useful indicator for assessing miscibility, with a difference of Δδ < 5 MPa^0.5^,^26^ suggesting miscibility. The group contribution method is a viable approach to estimate δ, and several methods are reported for δ calculation.^26−29^ In this study, the Hoftyzer and van Krevelen method was applied to determine the solubility parameters of PEF, PET, and PE (calculations are detailed in the Supporting Information). The solubility differences among PET/PEF, PET/PE, and PEF/PE binary systems were found to be 2.5, 14.47, and 12.04 MPa^0.5^, respectively. Since PET and PEF are essentially similar polyesters, they are expected to be miscible or exhibit a low δ difference, whereas PEF/PE and PET/PE binary blends show immiscibility.

Interfacial tension is a key factor in determining the morphology of multiphase polymer systems introducing wettability as a driving force for spreading or encapsulation of the system components.^30^ Wettability can be assessed using the spreading coefficient, a thermodynamic measure introduced by Harkins. For a ternary blend, the spreading coefficient of component *k* (λ_*ikj*_) can be defined as follows:^31^
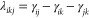
1where γ_*ij*_, γ_*ik*_, and γ_*jk*_ are the interfacial tensions between *i* and *j*, *i* and *k*, *j* and *k*, respectively. The interfacial tension between different components can be calculated using the geometric (Owen–Wendt) method:^32^
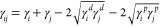
2Here, γ_*ij*_ is the interfacial tension between components *i* and *j*, γ_*i*_ and γ_*j*_ are their surface tensions, and γ_*i*_^*d*^, γ_*j*_^*d*^, γ_*i*_^p^, and γ_*j*_^*p*^are the dispersive and polar parts of their surface tensions, respectively. The interfacial tension between each two phases in the polyester/PE/compatibilizer ternary blends was determined based on the surface energies of the components at the processing temperature (Supporting Information). The results of the interfacial tension along with the spreading coefficient are provided in Table 2.

**Table 2:** Calculated Interfacial Tension and Spreading Coefficient

binary system	γ_*ii*_ (N/mm)		λ_*ikj*_
PET/PE	5.9	λ_PET–PE-*g*-MA-PE_	1.6
PET/PE-*g*-MA	2.4	λ_PE–PET-PE-*g*-MA_	–6.5
PET/SEBS-*g*-MA	3.2	λ_PET–PE-PE-*g*-MA_	–5.4
PE/PE-*g*-MA	1.9	λ_PET-SEBS-*g*-MA-PE_	2.2
PE/SEBS-*g*-MA	0.5	λ_PE–PET-SEBS-*g*-MA_	–8.6
		λ_PET–PE-SEBS-*g*-MA_	–3.3
PEF/PE	5.9	λ_PEF–PE-*g*-MA-PE_	2.5
PEF/PE-*g*-MA	2.1	λ_PE–PEF-PE-*g*-MA_	–6.5
PEF/SEBS-*g*-MA	3.3	λ_PEF–PE-PE-*g*-MA_	–5.2
PE/PE-*g*-MA	1.4	λ_PEF-SEBS-*g*-MA-PE_	2.2
PE/SEBS-*g*-MA	0.4	λ_PE–PEF-SEBS-*g*-MA_	–8.7
		λ_PEF–PE-SEBS-*g*-MA_	–3.0

**Figure 1:**
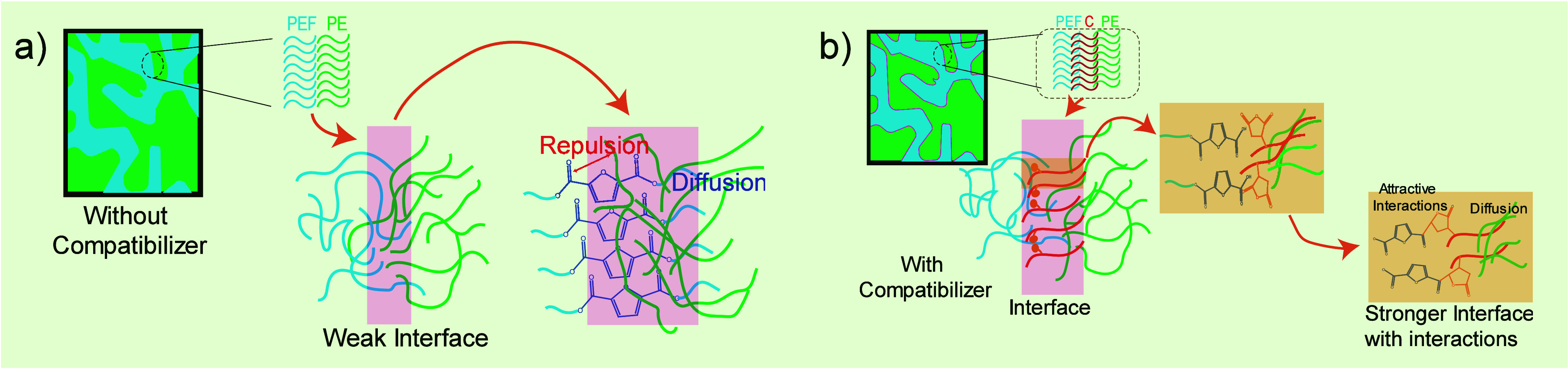
Predictive positioning of compatibilizer (C) in PEF/PE blends: (a) interface without compatibilizer and (b) interface with compatibilizer.

The calculation of the spreading coefficients for compatibilized PEF/PE blends (Table 2) resulted in λ_PEF-compatibilizer-PE_ > 0, whereas λ_PE–PEF-compatibilizer_ and λ_PEF–PE-compatibilizer_ < 0, indicating spreading of the compatibilizer between the polyester and PE phases due to higher interfacial tension between the two major components.^31^ Similar results were obtained for PET/PE compatibilized blends. Hence, irrespective of the morphological characteristics of the compatibilized system, both compatibilizers are anticipated to be positioned at the interface between the two phases, as depicted in Figure 1 for a cocontinuous system. This positioning is expected to decrease the interfacial tension between the blend’s components (discussed later).

### 3.2 Blends Characterization

#### 3.2.1 Miscibility, Morphology, and Interactions in Blends

Though the solubility differences indicate that PEF/PE and PET/PE are immiscible, morphological analysis provides further insights. In immiscible blends, compatibilizer refines and stabilizes the morphology and improves phase adhesion by decreasing the interfacial tension between components.^33^ Two common compatibilizers used in the polyester industry are PE-*g*-MA and SEBS-*g*-MA, and it would be practically important to understand how these compatibilizers will affect the morphology and functional properties of PEF and its blends.

**Figure 2:**
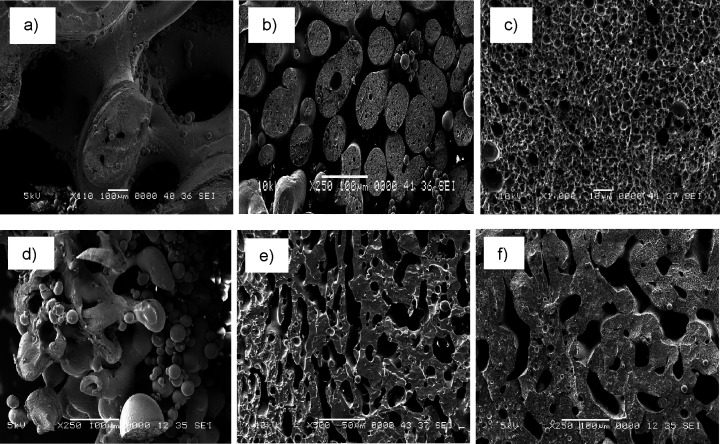
Morphology of (a) PEF/PE, (b) PEF/PE/PE-*g*-MA 10%, (c) PEF/PE/SEBS-*g*-MA 10%, (d) PET/PE, (e) PEF/PE/PE-*g*-MA 10%, and (f) PEF/PE/SEBS-*g*-MA 10%.

For SEM imaging, PEF and PET were extracted owing to the similar contrast between the polyesters and PE in imaging. Neat PEF/PE (Figure 2a) indicated poor compatibility as predicted by Δδ, where PEF formed incompatible large domains, whereas a semicocontinuous morphology was observed in the PET/PE blend (Figure 2d). In order to counterbalance the poor compatibility and reduce domain size, PEF/PE and PET/PE were compatibilized and examined.

Compatibilized PEF/PE and PET/PE blends exhibited very distinct morphologies (Figure 2b–f). For PEF/PE blends with PE-*g*-MA, a semicocontinuous morphology was observed at low compatibilizer concentration (Figure S1a). The domain size of each phase was smaller than that in the uncompatibilized PEF/PE blend (Figure 2a). Thinning PE domains with increasing PE-*g*-MA concentration led to a fibrillar morphology in the PEF/PE blend (Figure S1b and Figure 2b). At these concentrations, PEF remained continuous whereas PE mostly formed partially connected microfibers within the PEF matrix. It is worth mentioning here that the blends were compression molded before imaging resulting in the random orientation of the PE microfibers embedded in the PEF matrix.

Interestingly, a very different morphology was observed for PEF/PE compatibilized with SEBS-*g*-MA. Already at 1.5 wt % SEBS-*g*-MA (Figure S1c), the PEF/PE blend exhibited a significantly different morphology as compared to the uncompatibilized PEF/PE blend (Figure 2a). The PEF phase changed from an incompatible large domain to finely dispersed droplets in the continuous PE phase. A similar morphology was noticed in 50/50/20 PET/PE/SEBS-*g*-MA blends in a previous study, where the resulting morphology was attributed to the reduction in PET viscosity due to compatibilization.^34^ Increasing the SEBS-*g*-MA concentration to 5 wt % reduced PEF droplet size from micro size (0.9–34 μm) to micro- and nanoscale (220 nm–12 μm) (Figure S1d), which reduced further at 10 wt % SEBS-*g*-MA (Figure 2c) to 220 nm to 6 μm. This suggests that the compatibilizer effectively promotes finer dispersion through interfacial stabilization and reduction of interfacial tension. The mixture of nanoscale and microscale was also reported in a dispersed system of poly(ethylene-*co*-methyl acrylate)/poly(vinylidene fluoride) (PEMA/PVF) blend at lower composition of PVDA up to 30 wt %.^35^

However, despite the distinct morphologies of the polyester/polyolefin blends, it is interesting to notice that PE in all blends, excluding the PEF/PE/SEBS-*g*-MA blends, formed a multidomain structure as it showed small internal PEF domains known as the subphase. A similar observation was reported by Carté and Moet in the PET/PE system,^34^ which was attributed to the entrapment of some PET droplets within the PE melt during cooling. Since PET and PEF crystallize at a higher temperature compared to PE, they can establish a subphase within the PE phase. On the other hand, small spherical debris was noticed in the SEM images of some systems, which represents the accumulation of the extracted phase on the sample surface.

PE-*g*-MA compatibilized PET/PE blends exhibited a semicocontinuous morphology at a low compatibilizer concentration (Figure S1e), similar to the neat PET/PE blend. At 5 (Figure S1f) and 10 wt % of PE-*g*-MA (Figure 2e), the PET/PE blend is still fairly cocontinuous. A similar pattern of compatibility was observed for PET/PE blends with SEBS-*g*-MA. Though the neat PET/PE blend was also cocontinuous (Figure 2d), PET domain size reduced at 1.5 wt % SEBS-*g*-MA (Figure S1g) that decreased further at 5 wt %, leading to a dispersed PET within the PE matrix (Figure S1h), which is in line with the reported literature.^34^ A semicontinuous PET in continuous PE was observed at 10 wt % SEBS-*g*-MA (Figure 2f).

The evolution of morphology under extrusion is governed by a reduction in the particle size of the dispersed phase^36^ via mechanical drag and subsequently, refining toward smaller domain sizes and narrow particle size distribution. The second stage is characterized by the competition between coalescence and breakup processes and is controlled by the viscosity ratio (λ) and the capillary number (Ca),^37^ calculated as *Ca* = η_*m*_γ̇*R*/α, where η_*m*_is matrix viscosity, γ̇ is the shear rate, *R* is the droplet radius, and α is the interfacial tension.^32^ Under the influence of flow, fluid droplets are subjected to deformation and orientation by the stressed medium. The fluid droplets may deform into fibrils if the hydrodynamic forces outweigh the effects of interfacial tension.^37^ For Newtonian droplets under shear flow,^38^ λ < 0.7 causes the dispersed phase into small droplets; 0.7 < λ < 3.7 leads to stretching drops forming elongated threads; and beyond λ > 3.7, the formation of fibril structures becomes rare, as the critical capillary number in shear flow approaches infinity as λ approaches 3.7. On the other hand, in the viscoelastic system (polymers or blends), droplets can break up even at λ > 4 under extrusion conditions,^39^ whereas a fibrillar morphology with extended fibers can be observed at 1.92 < λ < 9.35.^37^Table 3 shows the complex viscosities of extruded neat polymers including PEF, PET, and PE, and also PEF, PE, and PE compatibilized with 10 wt % of the compatibilizers at the corresponding extrusion shear rate 5.53 s^–1^ (see the Supporting Information for estimation of this value and the rheological measurements procedure). The results reveal that the viscosities of both neat and compatibilized PEF and PET are significantly lower compared to those of neat and compatibilized PE processed at similar temperatures, resulting in a low viscosity ratio of PEF (PET)/PE blends in the range of 0.01–0.2. The low λ in the uncompatibilized of PEF/PE and PET/PE systems explains the observed coalesced morphologies in Figure 2a,d, since coalescence in uncompatibilized blends increases when the viscosity of the droplet is low and the concentration of the dispersed phase is high, whereas coalescence decreases at higher shear rates.^40^

**Table 3:** Complex Viscosity of Neat and Compatibilized PEF, PET, and PE at an Angular Frequency of 5.53 s^–1^

system	*T* °C	η Pa s	system	*T* °C	η Pa s
PEF	240	73.5	PET	270	239.1
PEF/PE-g-MA 10 wt %	240	32.4	PET/PE-g-MA 10 wt %	270	45.6
PEF/SEBS-g-MA 10 wt %	240	21.1	PET/SEBS-g-MA 10 wt %	270	129.6
PE	240	1552.3	PE	270	1152.0
PE/PE-g-MA 10 wt %	240	1077.5	PE/PE-g-MA 10 wt %	270	901.0
PE/SEBS-g-MA 10 wt %	240	1728.5	PE/SEBS-g-MA 10 wt %	270	1257.9

At a high concentration (10 wt %) of PE-g-MA, the presence of PE-g-MA led to an even smaller λ for the PEF/PE blend, falling within the range inducing droplet breakup. The droplet breakup is anticipated since the change in the viscosity of PE due to compatibilization is small, maintaining a comparable Ca to that of the uncompatibilized blend. However, the notable interaction between PEF and PE-g-MA, evidenced by the increased viscosity of the PEF/PE-g-MA blend in the low-frequency range (Figure S2a), suggests a significant reduction in the interfacial tension between the phases of the PEF/PE compatibilized blend. This reduction leads to a high capillary number (Ca), indicating the predominance of shear stress over interfacial stress, resulting in particle elongation^10,32^ and subsequent stress-induced fibrillation.^10^Figure 2c illustrates that the polymer with the highest viscosity, PE, tends to form fibers in PEF/PE/PE-g-MA blends at higher concentrations of PE-g-MA. While this observation contradicts some findings in the literature,^30^ it aligns with predictions from the cocontinuity models (Figure S3) that PEF remains the continuous phase as elaborated further below.

For PEF/PE/SEBS-g-MA blends, λ of PEF/PE compatibilized with 10 wt % of SEBS-g-MA was the smallest (0.01) justifying the dispersion of PEF in the PE matrix as evidenced by the SEM images. Generally, the formation of a dispersed phase of PEF in PEF/PE/SEB-g-MA x% blends might be attributed to the reduction in the interfacial tension due to compatibilization, leading to a Ca value laying in the range that enhances droplet deformation and breakup.^10^ However, Sundararaj and Macosko^41^ proposed that the reduction in drop size observed in a copolymer-compatibilized system is a result of inhibiting coalescence by the copolymer rather than reducing the interfacial tension. They also reported that reactive compatibilization was found to be more efficient in eliminating coalescence, even at high concentrations of the dispersed phase.

**Figure 3:**
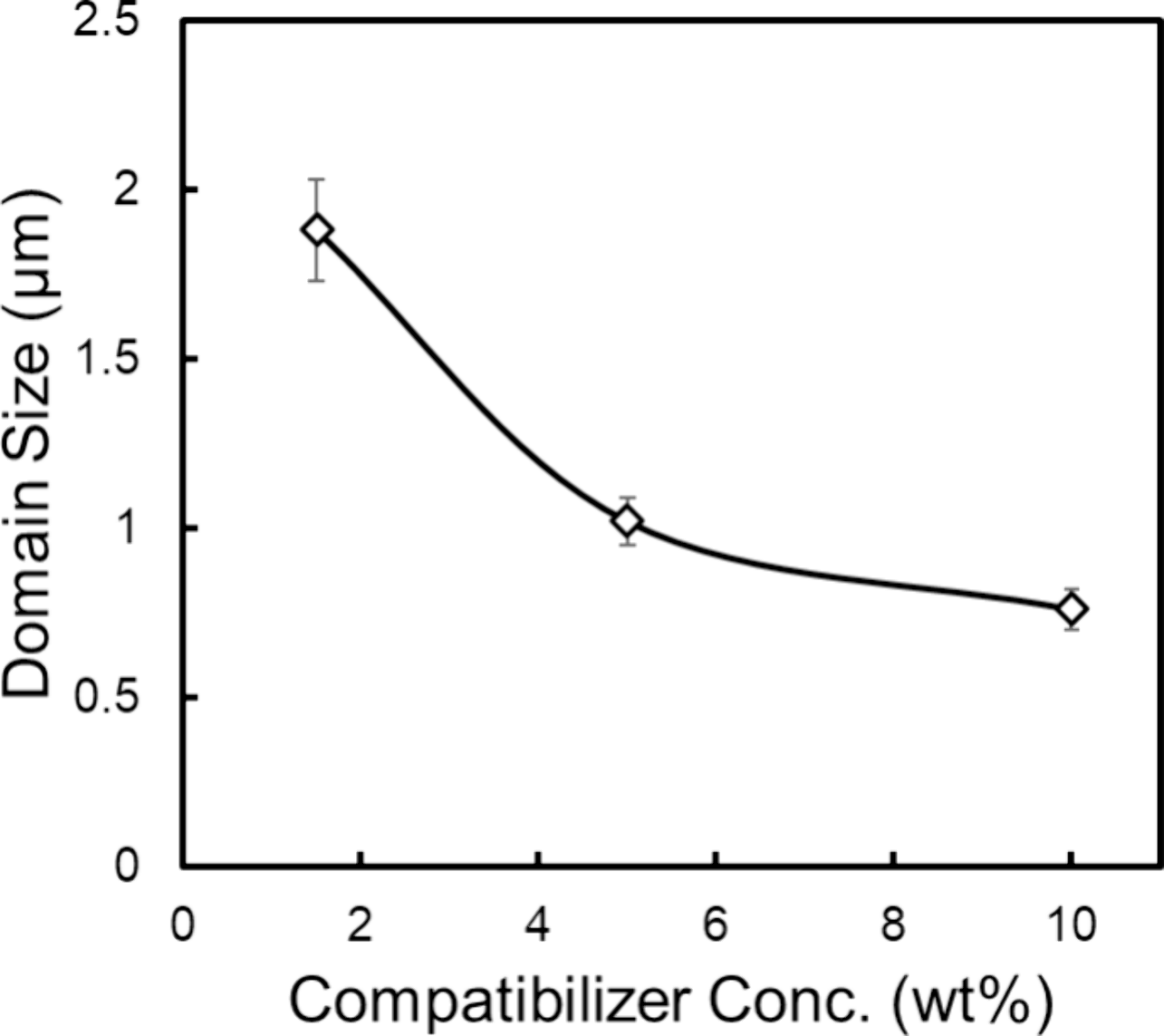
Effect of the compatibilizer concentration on the dispersed PEF particle size.

Since the interfacial tension is directly proportional to the domain size of the dispersed phase, it is appropriate to estimate interfacial saturation and the critical micelle concentration (CMC)—the concentration at which micelles begin to form—from a plot of domain size versus copolymer concentration.^42^ Below the CMC, the particle size reduction is linear with increasing copolymer content. The leveling off of particle size at higher block copolymer concentrations indicates interfacial saturation. Beyond this point, the addition of more copolymers may be inefficient, as it no longer modifies the interfacial region but instead leads to the formation of copolymer micelles within the homopolymer phases.^42^Figure 3 illustrates a sharp decrease in the dispersed phase dimensions with increasing the SEBS-g-MA concentration in PEF/PE from 1.5 to 5 wt %. A change in slope is noticed, indicating a gradual leveling off as the copolymer content is increased to 10 wt %. This concentration can be considered as the apparent CMC. Therefore, for SEBS-g-MA-compatibilized PEF/PE blends, adding more than 10 wt % of the compatibilizer is unlikely to result in further improvements in the blend morphology.

Low viscosity ratios were also observed in blends of uncompatibilized PET/PE and compatibilized PET/PE, indicating conditions inducing particle breakup. However, a cocontinuity was observed in all PET/PE blends except for PET/PE/SEBS-g-MA 5%, where PET was dispersed within the PE matrix. The development of a cocontinuous morphology can be understood by referring to the sheet formation mechanism in the initial stages of morphology development. Subsequent to sheet formation, interfacial disturbances on the sheet surface lead to sheet breakup, resulting in either a dispersed morphology or the presence of stable, elongated ribbons, thereby forming a cocontinuous structure.^43^ This suggests that in the compatibilized PET/PE blends, the interfacial reaction is comparatively weaker compared to the compatibilized PEF/PE blends, thereby stabilizing the elongated connected structure due to interfacial forces, resulting in a cocontinuous morphology.

In addition, cocontinuity is predicted in the vicinity of phase inversion in polymer blends. The phase inversion is defined as the blend composition in which the dispersed and matrix polymers are interchanged in a polymer blend. Phase inversion can be estimated using empirical relations such as those of Jordhamo et al.^44^ (later modified and called the Miles–Zurek model^45^), Ho et al.,^46^ Metelkin–Blekht,^47^ and Utracki and Shi.^39^ For the current study, experimental viscosity ratios were plotted versus the volume fraction ratios in order to assess the mentioned models (Figure S3). Interestingly, all blends exhibited volume fractions higher than the phase inversion composition prediction, thereby implying that PEF and PET are expected to be continuous phases. Although our experimental data do not align with the aforementioned models for phase inversion, it is important to note that these models have a limitation that they do not account for the influence of compatibilization on the blend’s morphology.

**Figure 4:**
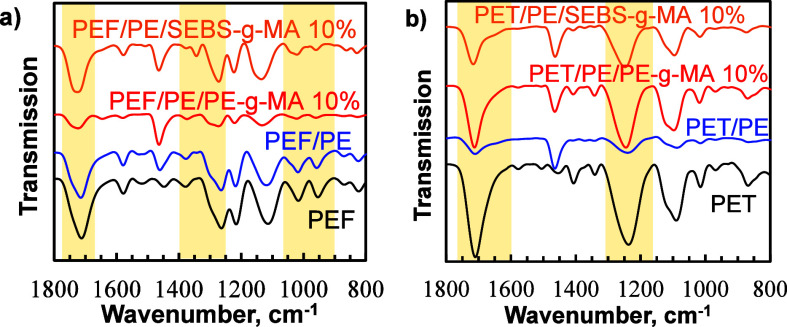
FTIR spectra of (a) PEF/PE blends and (b) PET/PE blends.

The changes in the blend morphology (Figure 2) further indicate that interactions play a key role in defining the resultant morphology in PEF/PE and PET/PE blends with both compatibilizers. A further insight into the chemical interactions was generated using FTIR (Figure 4). In general, it is reported that compatibilization with MA-containing compatibilizers in PET blends is associated with interactions between the anhydride group in the compatibilizer and the hydroxyl end group in PET.^48^ The FTIR spectrum of the uncompatibilized PEF/PE blend retained all characteristics of the blend components including the PEF peaks such as the carbonyl group (C=O at ∼1713 cm^–1^), 2,5-disubstituted furan heterocycles (peaks at 3123, 1578, 1262, 1016, 951, 820, and 753 cm^–1^), and ester bonds (−COO– at 1262 and 1116 cm^–1^)^9,49^ as well as the intensive peaks at 2912, 2844, and 1465 cm^–1^ referring to the stretching of C–H evidencing PE (Figure 4a). No apparent interactions in PEF/PE were observed, in agreement with the morphology observed by SEM and the prediction of immiscibility.

**Scheme 2:**
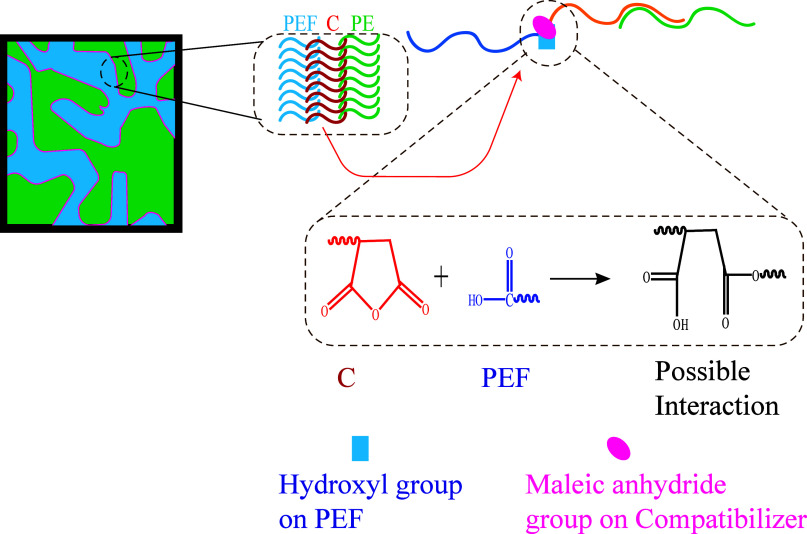
Predicted Interactions in Blend Components

At a concentration of 1.5 wt % of PE-*g*-MA in the PEF/PE blend, no changes were detected in the blend spectrum. However, as the concentration of PE-*g*-MA increased in the PEF/PE blends, notable shifts occurred in the peaks. Specifically, in PEF/PE/PE-*g*-MA blends containing 5 and 10 wt % of the compatibilizer, the peaks corresponding to the carbonyl group shifted to 1725 cm^–1^ and that of the ester bond to 1274 cm^–1^ (Figure 4a and Figure S4a). These shifts suggest potential interactions within the PEF/PE/PE-*g*-MA system, likely resulting from a reaction between the anhydride group of PE-*g*-MA and the hydroxyl group in PEF, leading to the formation of a PE-*g*-MA-PEF copolymer. This reaction is illustrated in Scheme 2 (also in Scheme S1) and aligns with findings from studies investigated PET/PE,^34^ polypropylene (PP) and poly(butylene terephthalate) (PBT),^50^ and polylactic acid (PLA)/SEBS^51^ blends. Similarly, in PEF/PE/SEBS-*g*-MA blends, peaks corresponding to carbonyl and ester groups shifted to higher frequencies (1725, 1274 cm^–1^, respectively), indicating possible chemical interactions (Figure 4a). The interactions are in agreement with the reaction of the anhydride group of SEBS-*g*-MA with hydroxyl groups in PEF, thereby forming a copolymer of SEBS-*g*-MA-PEF (see Scheme S1).^51^

Similarly, PET/PE retained all characteristic peaks of both PET and PE, indicating a lack of interactions between the two phases (Figure 4b). However, the peaks of the carbonyl (−C=O– at 1710 cm^–1^) and ester (−COO- at 1238 cm^–1^) groups shifted to higher frequencies (1715 and 1245 cm^–1^, respectively) in PET/PE/PE-*g*-MA blends indicating similar interactions as those observed in PEF/PE-*g*-MA between the anhydride of PE-*g*-MA and the hydroxyl group of PET (Figure 4b, Figure S4b). Similar reactions were observed in PET/PE/SEBS-*g*-MA (Figure 4b, Figure S4b), also depicted in Scheme 2 and Scheme S1.

In order to better understand the influence of the reaction between the anhydride and hydroxyl groups, samples containing polyesters (PEF or PET) were individually blended with compatibilizers (SEBS-*g*-MA and PE-*g*-MA). Samples compatibilized with 10 wt % compatibilizer (SEBS-*g*-MA or PE-*g*-MA) were prepared. The resultant materials were dissolved in HFIP followed by centrifugation (4000 rpm) to separate the undissolved fractions (Figure S6). After a complete drying under vacuum at 70 °C, the FTIR spectra of the undissolved fractions were determined. It is expected that the spectrum of these undissolved fractions will show the characteristic peaks of the compatibilizer, since both compatibilizers are nondissolving in HFIP. However, characteristic peaks of both PEF and PET existed in the spectrum of the undissolved fraction (Figure S5). This observation confirms the presence of polyester-MA copolymer as a result of the proposed reactions (Scheme 2). While it is expected that this reaction would increase the viscosity of the system^24,52^ due to the formation of new copolymers, compared to the viscosity of the extruded PEF; a noticeable reduction in the viscosity of the PEF/PE-*g*-MA blend in the high-frequency range, and of the PEF/SEBS-*g*-MA blend over the complete frequency range was observed. GPC testing revealed a sharp decrease in the molecular weight of PEF from 85 to 54 kg/mol when being blended with PE-*g*-MA, indicating a dilution effect caused by the compatibilizer (Table S7). For the PEF/SEBS-*g*-MA blend, the molecular weight could not be measured due to the highly turbid sample. However, the viscosity reduction in this system may be attributed to the relatively low molecular weight of the compatibilizer^53^ which dilutes the system and leads to a lower viscosity. A similar observation was reported by Khonakdar et al.^54^ when they studied the effect of polypropylene-graft-maleic anhydride (PP)-*g*-MA on PET/PP blends.

Similar trends in PET viscosity were observed (Table 3). However, the reduction in extruded PET viscosity upon introducing the compatibilizer was less pronounced compared to that of the PEF viscosity, suggesting a lower degree of interaction between PET and the compatibilizers. Conversely, the impact of the compatibilizers on the viscosity of PE was much smaller (Figure S2b,d) indicating that the reaction primarily occurs within the polyester phase.

#### 3.2.2 Thermal Properties of Blends

The thermal properties of PEF/PE and PET/PE blends were evaluated by DSC and TGA (the TGA procedure is explained in the Supporting Information). The DSC scan traces changes in the thermal behavior and polymer crystallinity due to blending or compatibilization.

**Figure 5:**
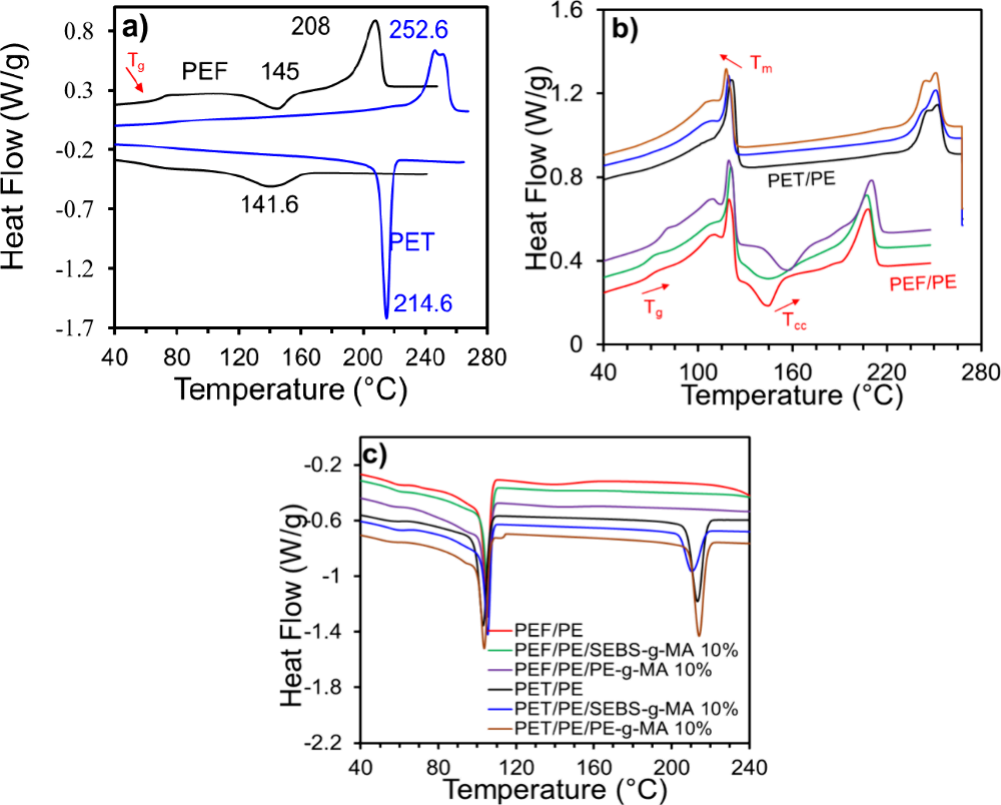
Thermal characteristics of PEF and PET blends: (a) comparison between PEF and PET; (b) heating scan on PEF (PET)/PE blends, and (c) cooling scan on PEF (PET)/PE blends (color codes are same in parts b and c).

The PEF was compared with PET in order to differentiate between the thermal behaviors of these two counterparts (Figure 5a). The neat as-received PEF exhibited a *T*_g_ of 85.5 °C (Figure S9), higher than that of as-received PET (75 °C). Compared to molded PEF, molded PET showed a split peak melting at 247.7 and 252.6 °C, indicating the presence of crystals with different lamellar thicknesses undergoing melting at slightly different temperatures.^55,56^ The higher crystallization temperature of PET (214.6 °C) is the most pertinent factor compared to the PEF crystallization temperature (141.6 °C).

There was no significant change observed in the melting behavior of uncompatibilized PET/PE and compatible blends (Figure 5b). The melting temperature (*T*_m_) of PET in these blends exhibited a slight increase in the *T*_m_ of PET phase compared to the pristine polyester, while the *T*_m_ of PE remained unchanged, except for a decrease of 3 °C at the highest concentration of PE-*g*-MA. However, the decrease in the crystallization temperature (*T*_c_) of both phases, particularly PET, suggests enhanced chain interaction and molecular chain entanglement between the two phases due to reactive extrusion, which hinders the crystallization process.^57−59^ On the other hand, in PEF/PE blends, the *T*_g_, cold crystallization temperature *T*_cc_, and *T*_m_ of PEF increased significantly with compatibilization with PE-*g*-MA, indicating considerable interactions in the presence of this compatibilizer. However, there was no noticeable change in the PE melting in PEF/PE blends, indicating that compatibilizers mainly affected the PEF. Upon cooling, PEF exhibited completely amorphous behavior in PEF/PE blends, while PE’s peak crystallization temperature, *T*_c_ decreased slightly by the compatibilizers, further supporting the hypothesis that crystallization was hindered by the compatibilization process. The changes in thermal properties of the blends are listed in Table S8.

The shifts observed in the glass transition temperature within polymer blends are primarily caused by miscibility, either partial or complete. In miscible systems, one *T*_g_ is typically observed, whereas in immiscible systems, two *T*_g_ values independent of the phase compositions can often be identified. However, despite the confirmed immiscibility of the blends in the current study, as evidenced by SEM, only the *T*_g_ of the polyester phase could be detected within the temperature range of the test since the reported *T*_g_ of polyethylene is situated at around −100 °C.^60^

**Figure 6:**
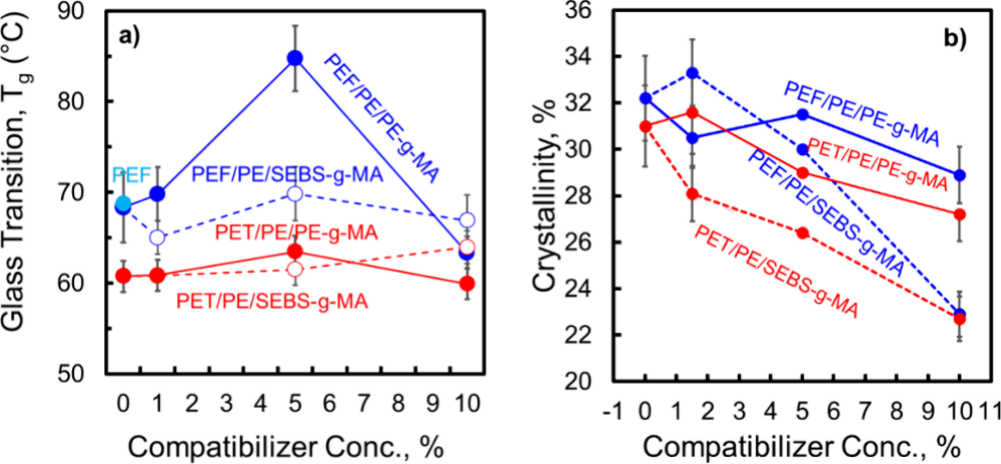
(a) Glass transition temperature (*T*_g_) of PEF and PET in their blends. (b) Crystallinity of PEF/PE and PET/PE blends.

In immiscible blends, with the introduction of a compatibilizer, a convergence in the distinct *T*_g_ values may occur.^61^ Moreover, variations in *T*_g_ may also originate from morphological alterations,^62^ chemical or physical interactions, or dilution effects. The interphase, i.e., the region of interpenetration, formed due to the chemical reaction or physical interaction usually results in alterations in the blend *T*_g_ due to segmental motions occurring within the interphase.^61^ In systems characterized by large domains resulting from immiscibility, no significant shift in *T*_g_ is anticipated. This phenomenon is evident in the PEF/PE blend (Figure 6a), where the *T*_g_ of PEF remains typical of that observed in the neat polymer.

For blends characterized by dispersed morphologies, where one component has a crystallization temperature higher than the *T*_g_ of the other component, changes in *T*_g_ are often observed. This phenomenon is attributed to the isotropic pressure exerted during crystallization, which can increase the *T*_g_ of the dispersed component, as seen in similar systems like polystyrene/ethylene blends.^62^ However, in PEF/PE/SEBS-*g*-MA blends, the high concentration of PEF (50 wt %) minimizes the impact of isotropic pressure, leading to only minor changes in *T*_g_. Additionally, the introduction of the compatibilizer could potentially enhance the change in *T*_g_ in a direction opposite to the effect of isotropic pressure, resulting in a minimum change in the *T*_g_ of PEF compared with that of the neat PEF.

*T*_g_ showed a significant increase in all PEF/PE blends compatibilized with PE-*g*-MA. The most notable change was observed at a compatibilizer concentration of 5 wt %, where the *T*_g_ of PEF in the neat blend (69.7 °C) rose to 81.0 °C. This increase occurred despite the fibrillar morphology developed in this blend, characterized by semiconnected PE fibers. The morphology suggests that PE has a limited impact on the thermal behavior of PEF, as the structure indicates that PEF surrounds PE, minimizing its effect on the *T*_g_ of the polyester phase. However, the significant rise in *T*_g_ could be attributed to interdiffusion into PEF chains, leading to a higher *T*_g_ due to restriction in the chain’s movement. Similar effects have been noted in other studies of reactive compatibilizers. For instance, Fredi et al. observed a 3 °C increase in the *T*_g_ of PLA when blended with 30% PEF and compatibilized using Joncryl ADR.^63^

For PET/PE blends, the *T*_g_ values of the polyester phase remained almost similar (Figure 6a). This can be explained by the cocontinuous morphology, where the amorphous regions of the polyester experience both compressive stresses and tension, depending on their contact with PE.^62^ These opposing forces lead to a minimal change in the PET *T*_g_.

The crystallinity of the PEF/PE blends (Figure 6b) as measured from XRD patterns (Figure S8b,c) exhibited comparable values for uncompatibilized and PE-*g*-MA-compatibilized blends, suggesting that the PE-*g*-MA penetrates the phases and acts as a nucleation agent, which balances the reaction effect on the crystallinity. A similar observation was noted in the PEF/PE/SEBS-*g*-MA 1.5% blend. However, a reduction in crystallinity occurred at higher concentrations of SEBS-*g*-MA, particularly at 10%, which showed a significant decrease. That might be attributed to the compatibilization as the product of the compatibilization reaction impedes the crystallization of the phases. Comparably, the crystallinity of PET/PE blends almost remained unaffected by compatibilization with PE-*g*-MA (Figure 6b) but gradually declined with the addition of SEBS-*g*-MA reaching its lowest at 10% of the SEBS-*g*-MA.

On the other hand, TGA results revealed that compatibilizing the PEF/PE and PET/PE blends enhances the blends thermal stability, indicating stronger interface due to better interfacial adhesion^64^ (Figure S11 and Table S9).

#### 3.2.3 Dynamic Mechanical Analysis (DMA)

DMA is a valuable tool for predicting the miscibility of polymeric systems. Typically, for an immiscible polymer blend, the tan δ curve exhibits two distinct damping peaks corresponding to the *T*_g_ values of the individual polymers. In contrast, for blends of two amorphous polymers, the presence of a single value, intermediate between those of the pure components, confirms the miscibility of the system.

**Figure 7:**
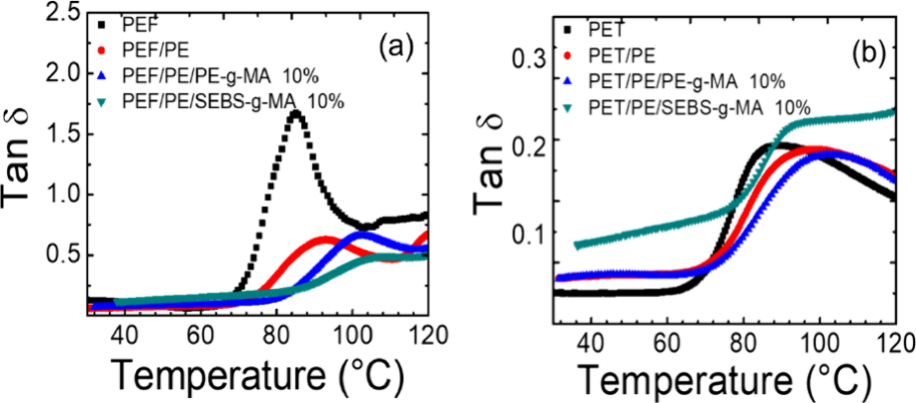
Tanδ of (a) PEF/PE blends and (b) PET/PE blends.

For pure PEF (Figure 7a), the tanδ curve shows a sharp increase in tan δ value at the *T*_g_ region, which aligns with the low entanglement density of PEF.^65^ In the uncompatibilized PEF/PE blend, a decrease in tan δ peak height is observed, attributed to a lower population of the amorphous region in this system.^65^ On the other hand, broader tan δ peaks are evident in PEF/PE blends compatibilized with PE-*g*-MA and SEBS-*g*-MA. This broadening indicates interactions between the components, along with a noticeable shift in T_g._^66^

Similarly, PET/PE (Figure 7b) blends showed a smaller shift in *T*_g_ compared with the neat polymers, consistent with observations from DSC analysis. However, the tan δ peaks in these blends also exhibited broadening, indicating interactions between the components in the system. This broadening reflects an increased complexity in molecular dynamics, further confirming the compatibilization effects.

**Figure 8:**
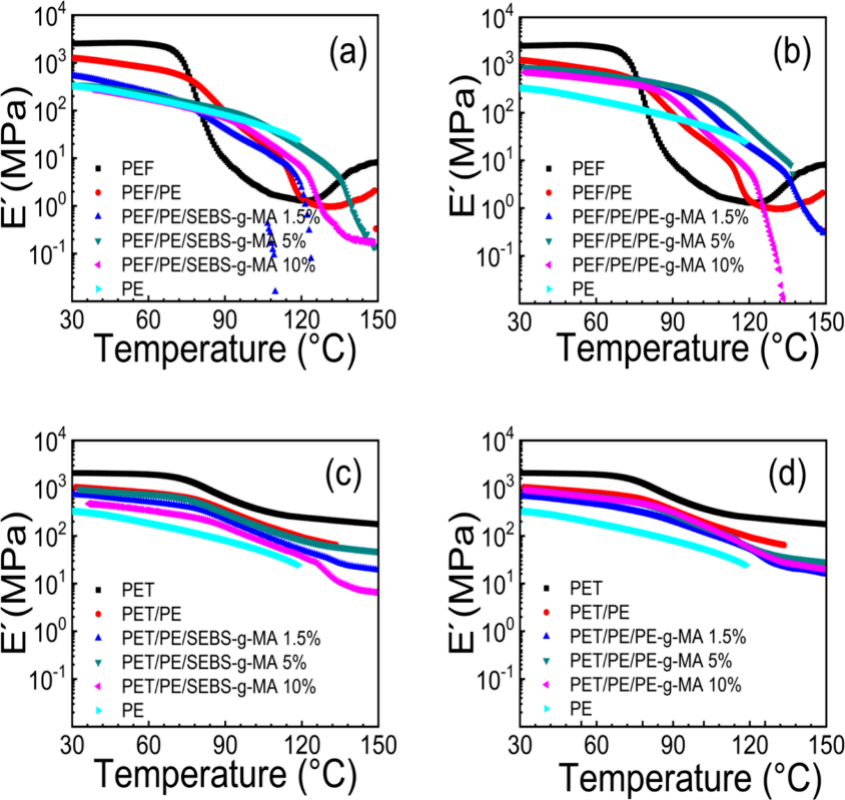
DMA Patterns for (a) PEF/PE/SEBS-*g*-MA blends, (b) PEF/PE/PE-*g*-MA blends, (c) PET/PE/SEBS-*g*-MA blends, and (d) PET/PE/PE-*g*-MA blends.

On the other hand, DMA results exhibited high rigidity in the pure PEF and PET, owing to their elevated moduli values (Figure 8). The storage modulus (*E*′) of PEF is higher than that of PET, attributed to the restricted chain mobility of the furan ring in PEF in comparison to the benzene ring in PET which results in increased stiffness and glass transition temperature (*T*_g_).^9^ The uncompatibilized blends exhibited higher moduli than the compatibilized systems as the lack of interaction between the polyester and polyolefin phases preserves greater stiffness in the blend. A sharp decrease in *E*′, along with the absence of a plateau region, was observed in PEF/PE/SEBS-*g*-MA blends. This phenomenon can be attributed to the dispersed morphology, where the PE (matrix phase) dominates the overall modulus pattern.^48^ Thereby, enhanced tensile toughness is expected in these blends, owing to the inherent toughness of PE.

Conversely, PEF/PE/PE-*g*-MA exhibited a high *E*′ comparable to that of uncompatibilized PEF/PE blends. Moreover, these compatibilized systems displayed an extended plateau region reflecting a greater contribution of PEF, indicating the cocontinuity of the blend phases. Similarly, the cocontinuity of PET blends was confirmed by the plateau region observed in the *E*′ patterns, indicating the contribution of both phases in the uncompatibilized and compatibilized blends. Interestingly, all systems compatible with 5% of the copolymers showed a higher modulus within their respective groups. This observation can be linked with the higher *T*_g_ observed in the thermograms of these blends, indicating higher interactions in comparison to the other systems.

#### 3.2.4 Mechanical Properties of Blends

The inherent brittleness of PEF poses limitations on its potential applications in the polymer market. Consequently, blending PEF with ductile polyolefins is promising in terms of enhancing the ductility and toughness of PEF. Based on the analysis of FTIR, SEM, and DSC data, it can be inferred that the observed interactions between the two phases may lead to anticipated enhancements in mechanical properties.

**Figure 9:**
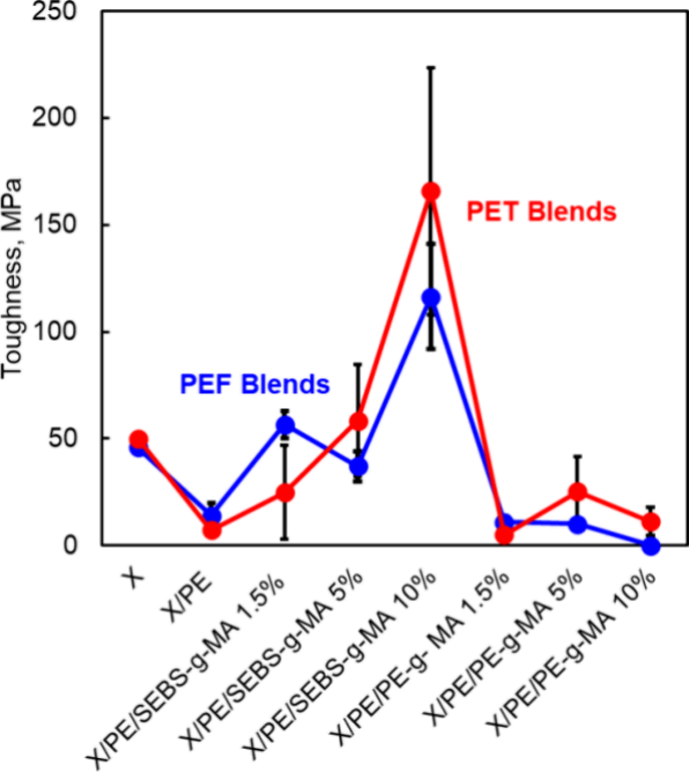
Tensile toughness of PEF/PE and PET/PE blends.

Furthermore, tensile testing results are presented in Table 4, Figure 9, and Figure S12. Analysis of the obtained tensile stress vs strain curves revealed that neat PEF exhibited the highest average tensile strength with 34 MPa. However, the incorporation of PE into the blend led to a decrease in tensile strength to 12.1 ± 2 MPa. Additionally, there was a deterioration in elongation at the break and tensile toughness, indicating the incompatibility of the blended phases characterized by large phase domains, resulting in a very poor mechanical performance.

**Table 4:** Tensile Properties of PEF/PE and PET/PE^a^

			**PEF/PE/SEBS-g-MA**				**PEF/PE/PE-g-MA**		
	PE*	**PEF**	**0**	**1.5**	**5**	**10**	**1.5**	**5**	**10**
YM	0.34 ± 0.0	1.9 ± 0	0.89 ± 0.0	0.43 ± 0.1	0.38 ± 0.0	0.29 ± 0.01	0.74 ± 0.2	0.71 ± 0.1	
σ	10.9 ± 3.6	34.7 ± 0	12.1 ± 0.1	9.7 ± 0.63	6.8 ± 0.84	7.4 ± 0.32	10.5 ± 1.6	9.1 ± 2.39	
ε	343 ± 43	2.4 ± 0	2.0 ± 0.43	7.8 ± 0.9	6.7 ± 1.0	19.3 ± 2.8	1.8 ± 0.2	1.9 ± 0.14	
T	2953 ± 675	46 ± 0	14.0 ± 6.1	56.7 ± 6.4	37.1 ± 7.1	116.5 ± 25	11.9 ± 3.0	10.2 ± 0.8	

			**PET/PE/SEBS-g-MA**				**PET/PE/PE-g-MA**		
		**PET**	**0**	**1.5**	**5**	**10**	**1.5**	**5**	**10**
YM		1.45 ± 0.0	0.71 ± 0.1	0.71 ± 0.1	0.71 ± 0.1	0.35 ± 0.07	0.76 ± 0	0.75 ± 0.0	0.71 ± 0.0
σ		27.7 ± 0.0	10.0 ± 3.0	10.8 ± 0.9	12.2 ± 2.7	10.0 ± 2.3	5.0 ± 0.0	13.7 ± 3.3	10.35 ± 5.0
ε		3.0 ± 0.0	2.0 ± 1.07	3.3 ± 2.4	5.8 ± 1.0	19.6 ± 4.5	1.2 ± 0.0	2.6 ± 1.8	2.2 ± 0.1
T		50 ± 0.0	7.2 ± 2.23	24.9 ± 22	58.5 ± 26	165.9 ± 58	5.0 ± 0.0	25.2 ± 16	11.3 ± 6.5

a YM, Young’s modulus (GPa); σ, tensile strength (Mpa); ε, strain at break (%); T, tensile toughness (MPa); *tested at an overhead speed of 5 mm/min, while the other samples tested at 2 mm/min.

The addition of small amounts of SEBS-*g*-MA as a compatibilizer induces the dispersion of PEF droplets within the PE matrix. This droplet-matrix morphology slightly improved elongation at break compared to the neat blend at low compatibilizer concentrations, as the PE matrix absorbs and dissipates the applied stress.^23^

At higher concentrations of SEBS-*g*-MA (10 wt %), smaller PEF droplets are observed, leading to a noticeable enhancement in elongation at break and corresponding tensile toughness. This blend showed an elongation at break 8 and 10 times higher than that of the pure PEF and uncompatibilized PEF/PE, respectively, and tensile toughness 2.5 and 8-fold higher than that of neat PEF and uncompatibilized PEF/PE, respectively. This improvement is attributed to the greater ability of the PE matrix to stretch during tensile testing, resulting in a higher elongation and increased toughness. However, a decrease in the elastic modulus is noticed in those blends. The reason behind that is that the SEBS-*g*-MA elastomer wraps around rigid PEF particles, forming a core–shell structure (PEF core, SEBS-*g*-MA shell). Some SEBS-*g*-MA chains mix with the PEF core, softening the overall structure and reducing the modulus.^67^ In comparison, a study on a 50/50 wt/wt blend of PEF and poly(butylene succinate) (PBS)^68^ showed that the developed blend exhibited less than a 10% improvement in elongation at break compared to neat PEF, while the tensile strength dropped to 26.2 MPa from 56.5 MPa in neat PEF. Nevertheless, the blend’s tensile strength remained significantly higher than that of neat PBS, which measured 2.5 MPa, due to the dominance of PEF as the matrix phase. The improved ductility of PEF noticed in this study due to the reactive compatibilization is consistent with findings from other studies, where PEF/polyamides 11 (PA11) blends were reactively compatibilized using Joncryl.^17^ The addition of 1.5 phr Joncryl to 80/20 wt/wt PEF/PA11 blend significantly improved elongation at break to 90.1% compared to 3.6% for neat PEF, whereas tensile strength decreased to 72.2 MPa in the blend compared to 84.5 MPa for the neat PEF. Another study^63^ showed that the addition of 1 phr of Joncryl to 97/3 wt/wt PLA/PEF blend increased the elongation at break to 7.1% (103% higher than neat PLA), yet it reduced the elastic modulus to 3.2 GPa which is 11% lower than that of neat PLA.

In contrast, for PEF/PE blends compatibilized with PE-*g*-MA, which exhibit a fibrillar morphology with PE forming fibrils, deteriorated mechanical performance is observed at 1.5 and 5 wt % compatibilizer. This behavior is due to the brittle nature of the PEF matrix, which limits elongation and consequently reduces toughness. Additionally, the random orientation of the fibrils in the blend does not facilitate improved stretchability compared with the uncompatibilized blend. It is worth mentioning that the specimens of PEF/PE/PE-*g*-MA-10% could not be successfully produced due to growing cracks during the sample’s cutting process. However, this phenomenon can also be explained by the random orientation of the fibers due to the compression molding process and requires further investigation in the future.

On the other hand, neat PET showed a lower tensile strength and lower elastic modulus (Figure S12) compared to neat PEF in line with the reported literature. However, PET blends showed trends of mechanical properties almost similar to those of PEF blends (Figure S12). The uncompatibilized PET/PE blends exhibited poor mechanical performance due to the presence of large, immiscible polymer domains. The addition of SEBS-*g*-MA progressively improved the morphology and mechanical properties progressively. At 5 wt % compatibilizer, the morphology transitions to a dispersed structure with PET-forming droplets that increase the elongation at break, while at 10 wt %, a cocontinuous morphology is observed. The latter demonstrates significant mechanical property enhancements, which is in agreement with results reported earlier in the literature,^69^ as the tensile toughness increased by 3, and 23 folds compared to that of the neat PET polymer, and uncompatibilized PET/PE blend, respectively. That can be attributed to better stress distribution within the connected phases, hence enhancing the toughness.^58^

Interestingly, in blends exhibiting similar cocontinuous morphologies due to PE-*g*-MA addition, deteriorated mechanical properties are reported. This decline is attributed to the higher crystallinity of blends compared to those compatibilized with SEBS-*g*-MA, which reduces the flexibility and toughness of the material.^70^

**Figure 10:**
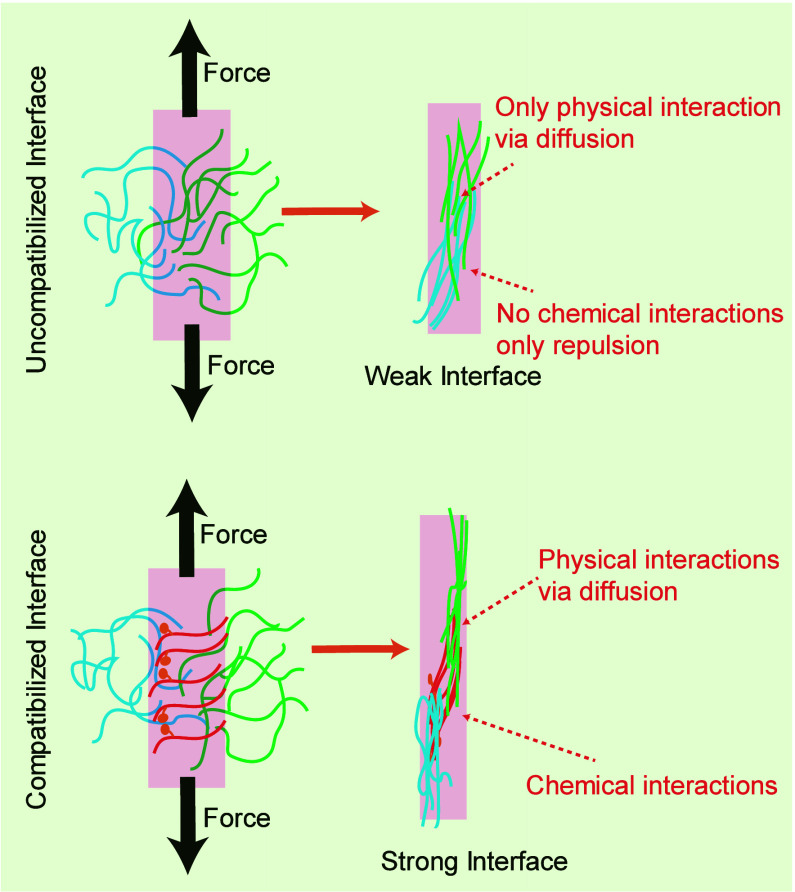
Toughening mechanism of the compatibilized blend under tensile force.

The toughening mechanism that led to notable enhancements in ductility and toughness in PEF/PE and PET/PE blends upon incorporation of SEBS-g-MA can closely be linked to specific morphological changes induced by the compatibilizer. SEBS-g-MA reduces the interfacial tension between polyester and PE due to the reaction of maleic anhydride groups in SEBS-g-MA with hydroxyl groups in polyester, enhancing adhesion between polyester and PE phases (Figure 10). This resulted in smaller and more uniformly distributed PEF domains while it led to a cocontinuous morphology in PET/PE. This variation in the morphology and microstructure formation depends on the concentration of hydroxyl groups available for reaction with the MA group.^71^ The improved adhesion facilitates stress transfer across the interface. Additionally, the copolymer resides at the interface between polyester and PE phases and forms a shell layer covering the dispersed PEF particle in the PEF/PE blend, leading to a core–shell structure, which further causes shear banding during crack propagation.^57,67^

#### 3.2.5 Sustainable Aspects of PEF/PE Blends

In the global pursuit toward sustainable materials and processes, PEF/PE blends represent a step toward eco-friendly alternatives. Polyethylene furanoate (PEF), derived from renewable resources, is a carbon-negative material, making it a more sustainable option compared to conventional petroleum-based polyethylene terephthalate (PET). However, the use of polyethylene (PE), a petroleum-derived polymer, reduces the overall sustainability of the blends. Replacing PE with biopolyethylene (bio-PE) could further lower the carbon footprint, offering a more environmentally friendly solution. Furthermore, the recyclability of both PEF and PE is advantageous in terms of promoting circular economy practices.

From a processing standpoint, the extrusion method used to produce the blend is energy-intensive. Despite this issue, extrusion is a more sustainable alternative compared to solution blending as it avoids the use of environmentally hazardous solvents. Nevertheless, the molding stage, which requires high temperatures, also contributes significantly to energy consumption. To mitigate the environmental impact of such processes, integrating carbon capture and storage (CCS) technologies could be effective in reducing the carbon footprint associated with manufacturing these blends.

This approach underscores the importance of combining material innovation with process optimization to achieve truly sustainable solutions. By addressing both material selection and processing impacts, the development of PEF-based polymer blends can align with global sustainability goals while minimizing environmental harm.

## 4 Conclusions

This study concludes the first comprehensive report on developing blends of biorenewable PEF with synthetic polyolefins. Due to the inherent incompatibility of PEF and polyolefins, two conventional compatibilizers were utilized to increase the adhesion between the polyester and polyethylene phases. The compatibilizer type and composition exhibited significant effects on the developed morphology in the blends. The SEBS-g-MA resulted in a dispersed morphology in PEF/PE with PEF being the dispersed phase, while PE-g-MA led to a fibrillar morphology for the same blend. Moreover, PE-g-MA compatibilizer promoted a cocontinuous morphology in PET/PE blends at various compatibilizer compositions, while SEBS-g-MA contributed to dispersed and cocontinuous morphologies for PET/PE at compatibilizer concentrations of 5 and 10 wt %, respectively. The reaction between the anhydride groups of the compatibilizers and the end hydroxyl group in the polyester phase contributed to the various changes in the morphology and physical properties of the blends

It was found that the elongation at the break increased with increasing SEBS-g-MA compatibilizer concentration. At 10% SEBS-g-MA, the elongation at break increased by 800%, and the tensile toughness enhanced by 250% as compared to those of the neat PEF due to the compatibilization effect. On the other hand, PE-g-MA had no effect on the elongation at break as well as the tensile toughness of the blends. It is important to highlight that these improvements can similarly be attained through the substitution of conventional polyethylene with biopolyethylene, thereby achieving a fully biobased blend with enhanced properties.

## Funding Resources

The research was funded through UAEU Program for Advanced Research (UPAR) grant number 12N002.

## Supporting Information Available

Sample compositions, prediction of polymer (im)miscibility, calculation of individual solubility parameters, free surface energy measurement and interfacial tension calculations, morphologies of the compatibilized blends, shear rate calculation, cocontinuity models used, FTIR results, XRD results, effects of the processing conditions on PEF properties, TGA results, DMA test, and mechanical properties (PDF)

## References

[ref1] Sousa A. F.; Vilela C.; Fonseca A. C.; Matos M.; Freire C. S. R.; Gruter G.-J. M.; Coelho J. F. J.; Silvestre A. J. D. Biobased polyesters and other polymers from 2,5-furandicarboxylic acid: a tribute to furan excellency. Polym. Chem. 2015, 6, 5961–5983. 10.1039/C5PY00686D.

[ref2] Awaja F.; Pavel D. Recycling of PET. Eur. Polym. J. 2005, 41, 1453–1477. 10.1016/j.eurpolymj.2005.02.005.

[ref3] Assadi R.; Colin X.; Verdu J. Irreversible structural changes during PET recycling by extrusion. Polymer. 2004, 45, 4403–4412. 10.1016/j.polymer.2004.04.029.

[ref4] Burgess S. K.; Kriegel R. M.; Koros W. J. Carbon Dioxide Sorption and Transport in Amorphous Poly(ethylene furanoate). Macromolecules. 2015, 48, 2184–2193. 10.1021/acs.macromol.5b00333.

[ref5] Knoop R. J. I.; Vogelzang W.; van Haveren J.; van Es D. S. High molecular weight poly(ethylene-2,5-furanoate); critical aspects in synthesis and mechanical property determination. J. Polym. Sci., Part A: Polym. Chem. 2013, 51, 4191–4199. 10.1002/pola.26833.

[ref6] Burgess S. K.; Karvan O.; Johnson J. R.; Kriegel R. M.; Koros W. J. Oxygen sorption and transport in amorphous poly(ethylene furanoate). Polymer. 2014, 55, 4748–4756. 10.1016/j.polymer.2014.07.041.

[ref7] Burgess S. K.; Mikkilineni D. S.; Yu D. B.; Kim D. J.; Mubarak C. R.; Kriegel R. M.; Koros W. J. Water sorption in poly(ethylene furanoate) compared to poly(ethylene terephthalate). Part 2: Kinetic sorption. Polymer. 2014, 55, 6870–6882. 10.1016/j.polymer.2014.10.065.

[ref8] Sousa A. F.; Patrício R.; Terzopoulou Z.; Bikiaris D. N.; Stern T.; Wenger J.; Loos K.; Lotti N.; Siracusa V.; Szymczyk A.; Paszkiewicz S.; Triantafyllidis K. S.; Zamboulis A.; Nikolic M. S.; Spasojevic P.; Thiyagarajan S.; van Es D. S.; Guigo N. Recommendations for replacing PET on packaging, fiber, and film materials with biobased counterparts. Green Chem. 2021, 23, 8795–8820. 10.1039/D1GC02082J.

[ref9] Burgess S. K.; Leisen J. E.; Kraftschik B. E.; Mubarak C. R.; Kriegel R. M.; Koros W. J. Chain Mobility, Thermal, and Mechanical Properties of Poly(ethylene furanoate) Compared to Poly(ethylene terephthalate). Macromolecules. 2014, 47, 1383–1391. 10.1021/ma5000199.

[ref10] Utracki L. A.; Mukhopadhyay P.; Gupta R. K.; Polymer Blends: Introduction. In Polymer Blends Handbook; Utracki L. A.; Wilkie C. A., Ed.; Springer: Netherlands: Dordrecht, 2014; pp 3–170.

[ref11] Bai L.; He S.; Fruehwirth J. W.; Stein A.; Macosko C. W.; Cheng X. Localizing graphene at the interface of cocontinuous polymer blends: Morphology, rheology, and conductivity of cocontinuous conductive polymer composites. J. Rheol. 2017, 4, 575–587. 10.1122/1.4982702.

[ref12] Salzano de Luna M.; Filippone G. Effects of nanoparticles on the morphology of immiscible polymer blends – Challenges and opportunities. Eur. Polym. J. 2016, 79, 198–218. 10.1016/j.eurpolymj.2016.02.023.

[ref13] Poulopoulou N.; Kasmi N.; Bikiaris D. N.; Papageorgiou D. G.; Floudas G.; Papageorgiou G. Z. Sustainable Polymers from Renewable Resources: Polymer Blends of Furan-Based Polyesters. Macromol. Mater. Eng. 2018, 303, 1800153–1800160. 10.1002/mame.201800153.

[ref14] Poulopoulou N.; Nikolaidis G. N.; Efstathiadou V. L.; Kapnisti M.; Papageorgiou G. Z. Blending as a process for controlling the properties of poly(ethylene 2,5-furandicarboxylate) (PEF): Fully biobased PEF/PBF blends. Polymer. 2023, 266, 125615–125627. 10.1016/j.polymer.2022.125615.

[ref15] Papageorgiou D. G.; Tsetsou I.; Ioannidis R. O.; Nikolaidis G. N.; Exarhopoulos S.; Kasmi N.; Bikiaris D. N.; Achilias D. S.; Papageorgiou G. Z. A New Era in Engineering Plastics: Compatibility and Perspectives of Sustainable Alipharomatic Poly(ethylene terephthalate)/Poly(ethylene 2,5-furandicarboxylate) Blends. Polymers (Basel). 2021, 13, 1070–1088. 10.3390/polym13071070. 33805314 PMC8038036

[ref16] Paszkiewicz S.; Irska I.; Piesowicz E. Environmentally Friendly Polymer Blends Based on Post-Consumer Glycol-Modified Poly(Ethylene Terephthalate) (PET-G) Foils and Poly(Ethylene 2,5-Furanoate) (PEF): Preparation and Characterization. Materials. 2020, 13, 2673–2690. 10.3390/ma13122673. 32545434 PMC7345711

[ref17] Yang Y.; Tian A.-P.; Fang Y.-J.; Wang J.-G.; Zhu J. Improvement in Toughness of Poly(ethylene 2,5-furandicarboxylate) by Melt Blending with Bio-based Polyamide11 in the Presence of a Reactive Compatibilizer. Chin. J. Polym. Sci. 2020, 38, 1099–1106. 10.1007/s10118-020-2449-z.

[ref18] Fredi G.; Dorigato A.; Dussin A.; Xanthopoulou E.; Bikiaris D. N.; Botta L.; Fiore V.; Pegoretti A. Compatibilization of Polylactide/Poly (ethylene 2, 5-furanoate)(PLA/PEF) Blends for Sustainable and Bioderived Packaging. Molecules. 2022, 27, 6371 10.3390/molecules27196371. 36234907 PMC9572422

[ref19] Fabris C.; Perin D.; Fredi G.; Rigotti D.; Bortolotti M.; Pegoretti A.; Xanthopoulou E.; Bikiaris D. N.; Dorigato A. Improving the Wet-Spinning and Drawing Processes of Poly(lactide)/Poly(ethylene furanoate) and Polylactide/Poly(dodecamethylene furanoate) Fiber Blends. Polymers. 2022, 14, 2910–2031. 10.3390/polym14142910. 35890686 PMC9322962

[ref20] Wang B.; Wei C.; Li C.; Sang L.; Wang L.; Wei Z.; Qi M. Incorporation of Poly(ethylene 2,5-furanoate) into Poly(butylene adipate-co-terephthalate) toward Sustainable Food Packaging Films with Enhanced Strength and Barrier Properties. ACS Sustain. Chem. Eng. 2023, 11, 597–606. 10.1021/acssuschemeng.2c05221.

[ref21] Yu Z.; Zhou J.; Cao F.; Zhang Q.; Huang K.; Wei P. Characterization and Thermal Properties of Bio-Based Poly(Ethylene 2,5-Furan Dicarboxylate). J. Macromol. Sci., Part B 2016, 55, 1135–1145. 10.1080/00222348.2016.1238335.

[ref22] Mengual A.; Juárez D.; Balart R.; Ferrándiz S. PE-g-MA, PP-g-MA and SEBS-g-MA compatibilizers used in material blends. Procedia Manuf. 2017, 13, 321–326. 10.1016/j.promfg.2017.09.083.

[ref23] Dobrovszky K.; Ronkay F. Effects of SEBS-g-MA on Rheology, Morphology and Mechanical Properties of PET/HDPE Blends. Int. Polym. Proc. 2015, 30, 91–99. 10.3139/217.2970.

[ref24] Dobrovszky K.; Ronkay F. Investigation of compatibilization effects of SEBS-g-MA on polystyrene/polyethylene blend with a novel separation method in melted state. Polym. Bull. 2016, 73, 2719–2739. 10.1007/s00289-016-1618-2.

[ref25] Abdul Wahab M. K.; Ismail H.; Othman N. Compatibilization Effects of PE-g-MA on Mechanical, Thermal and Swelling Properties of High Density Polyethylene/Natural Rubber/Thermoplastic Tapioca Starch Blends. Polym. Plast. Technol. Eng. 2012, 51, 298–303. 10.1080/03602559.2011.639331.

[ref26] Van Krevelen D. W.; Te Nijenhuis K.; Cohesive Properties and Solubility. In Properties of Polymers, Their Correlation with Chemical Structure, Their Numerical Estimation and Prediction from Additive Group Contributions, 4th ed.; Van Krevelen D. W.; Te Nijenhuis K., Ed.; Elsevier: Amsterdam, 2009; pp 189–227.

[ref27] Small P. A. Some factors affecting the solubility of polymers. J. Appl. Chem. 1953, 3, 71–80. 10.1002/jctb.5010030205.

[ref28] Hoy K. L. New values of the solubility parameters from vapor pressure data. J. Paint Technol. 1970, 42, 76–118.

[ref29] Hoy K. L.; The Hoy tables of solubility parameters. South Charleston, WV: Union Carbide Corp., Solvents & Coatings Materials, Research & Development Dept.; 1985.

[ref30] Shokoohi S.; Arefazar A. A review on ternary immiscible polymer blends: morphology and effective parameters. Polym. Adv. Technol. 2009, 20, 433–447. 10.1002/pat.1310.

[ref31] Virgilio N.; Marc-Aurèle C.; Favis B. D. Novel Self-Assembling Close-Packed Droplet Array at the Interface in Ternary Polymer Blends. Macromolecules. 2009, 42, 3405–3416. 10.1021/ma802544q.

[ref32] Willemse R. C.; Posthuma de Boer A.; van Dam J.; Gotsis A. D. Co-continuous morphologies in polymer blends: the influence of the interfacial tension. Polymer. 1999, 40, 827–834. 10.1016/S0032-3861(98)00307-3.

[ref33] Utracki L. A. Compatibilization of Polymer Blends. Can. J. Chem. Eng. 2002, 80, 1008–1016. 10.1002/cjce.5450800601.

[ref34] Carté T. L.; Moet A. Morphological origin of super toughness in poly(ethylene terephthalate)/polyethylene blends. J. Appl. Polym. Sci. 1993, 48, 611–624. 10.1002/app.1993.070480405.

[ref35] Remanan S.; Ghosh S.; Das T. K.; Das N. C. Nano to microblend formation in poly(ethylene-co-methyl acrylate)/ poly(vinylidene fluoride) blend and investigation of its anomalies in rheological properties. Nano-Struct. Nano-Objects. 2020, 23, 100487–100501. 10.1016/j.nanoso.2020.100487.

[ref36] Covas J. A.; Carneiro O. S.; Maia J. M. Monitoring the Evolution of Morphology of Polymer Blends Upon Manufacturing of Microfibrillar Reinforced Composites. Int. J. Polym. Mater. Polym. Biomater. 2001, 50, 445–467. 10.1080/00914030108035119.

[ref37] Hong J. S.; Ahn K. H.; Lee S. J. Strain hardening behavior of polymer blends with fibril morphology. Rheol. Acta 2005, 45, 202–208. 10.1007/s00397-005-0015-9.

[ref38] Rumscheidt F. D.; Mason S. G. Particle motions in sheared suspensions XI. Internal circulation in fluid droplets (experimental). J. Colloid Sci. 1961, 16, 210–237. 10.1016/0095-8522(61)90002-2.

[ref39] Utracki L.; Shi Z. Development of polymer blend morphology during compounding in a twin-screw extruder. Part I: Droplet dispersion and coalescence—a review. Polym. Eng. Sci. 1992, 32, 1824–1833. 10.1002/pen.760322405.

[ref40] Lyu S.; Bates F. S.; Macosko C. W. Coalescence in polymer blends during shearing. AlChE J. 2000, 46, 229–238. 10.1002/aic.690460203.

[ref41] Sundararaj U.; Macosko C. W. Drop Breakup and Coalescence in Polymer Blends: The Effects of Concentration and Compatibilization. Macromolecules. 1995, 28, 2647–2657. 10.1021/ma00112a009.

[ref42] Thomas S.; Prud′homme R. E. Compatibilizing effect of block copolymers in heterogeneous polystyrene/poly(methyl methacrylate) blends. Polymer. 1992, 33, 4260–4268. 10.1016/0032-3861(92)90266-Y.

[ref43] Pötschke P.; Paul D. R. Formation of Co-continuous Structures in Melt-Mixed Immiscible Polymer Blends. J. Macromol. Sci., Part C 2003, 43, 87–141. 10.1081/MC-120018022.

[ref44] Jordhamo G.; Manson J.; Sperling L. Phase continuity and inversion in polymer blends and simultaneous interpenetrating networks. Polym. Eng. Sci. 1986, 26, 517–524. 10.1002/pen.760260802.

[ref45] Miles I. S.; Zurek A. Preparation, structure, and properties of two-phase co-continuous polymer blends. Polym. Eng. Sci. 1988, 28, 796–805. 10.1002/pen.760281205.

[ref46] Ho R.; Wu C.; Su A. Morphology of plastic/rubber blends. Polym. Eng. Sci. 1990, 30, 511–518. 10.1002/pen.760300903.

[ref47] Metelkin V.; Blekht V. Formation of a continuous phase in heterogeneous polymer mixtures. Colloid J. USSR. 1984, 46, 425–429.

[ref48] Araujo L. M. G.; Morales A. R. Compatibilization of recycled polypropylene and recycled poly (ethylene terephthalate) blends with SEBS-g-MA. Polímeros. 2018, 28, 84–91. 10.1590/0104-1428.03016.

[ref49] Papageorgiou G. Z.; Tsanaktsis V.; Bikiaris D. N. Synthesis of poly(ethylene furandicarboxylate) polyester using monomers derived from renewable resources: thermal behavior comparison with PET and PEN. Phys. Chem. Chem. Phys. 2014, 16, 7946–7958. 10.1039/C4CP00518J. 24647534

[ref50] Sun Y.; Hu G.-H.; Lambla M.; Kotlar H. K. In situ compatibilization of polypropylene and poly(butylene terephthalate) polymer blends by one-step reactive extrusion. Polymer. 1996, 37, 4119–4127. 10.1016/0032-3861(96)00229-7.

[ref51] Sangeetha V. H.; Varghese T. O.; Nayak S. K. Toughening of polylactic acid using styrene ethylene butylene styrene: Mechanical, thermal, and morphological studies. Polym. Eng. Sci. 2016, 56, 669–675. 10.1002/pen.24293.

[ref52] Lin X.; Qian Q.; Xiao L.; Chen Q.; Huang Q.; Zhang H. Influence of Reactive Compatibilizer on the Morphology, Rheological, and Mechanical Properties of Recycled Poly(Ethylene Terephthalate)/Polyamide 6 Blends. J. Macromol. Sci., Part B 2014, 53, 1543–1552. 10.1080/00222348.2014.946840.

[ref53] Denac M.; Musil V.; Šmit I. Polypropylene/talc/SEBS (SEBS-g-MA) composites. Part 2. Mechanical properties. Compos. Part A Appl. Sci. Manuf. 2005, 36, 1282–1290. 10.1016/j.compositesa.2005.01.011.

[ref54] Khonakdar H. A.; Jafari S. H.; Mirzadeh S.; Kalaee M. R.; Zare D.; Saeb M. R. Rheology-morphology correlation in PET/PP blends: Influence of type of compatibilizer. J. Vinyl. Addit. Technol. 2013, 19, 25–30. 10.1002/vnl.20318.

[ref55] Fredi G.; Karimi Jafari M.; Dorigato A.; Bikiaris D. N.; Checchetto R.; Favaro M.; Brusa R. S.; Pegoretti A. Multifunctionality of Reduced Graphene Oxide in Bioderived Polylactide/Poly(Dodecylene Furanoate) Nanocomposite Films. Molecules. 2021, 26, 2938 10.3390/molecules26102938. 34063331 PMC8155896

[ref56] Kourtidou D.; Grigora M. E.; Tzetzis D.; Bikiaris D. N.; Chrissafis K. Graphene Nanoplatelets&rsquo; Effect on the Crystallization, Glass Transition, and Nanomechanical Behavior of Poly(ethylene 2,5-furandicarboxylate) Nanocomposites. Molecules. 2022, 27, 6653–6673. 10.3390/molecules27196653. 36235190 PMC9571983

[ref57] Zytner P.; Wu F.; Misra M.; Mohanty A. K. Toughening of Biodegradable Poly(3-hydroxybutyrate-co-3-hydroxyvalerate)/Poly(ε-caprolactone) Blends by In Situ Reactive Compatibilization. ACS Omega. 2020, 5, 14900–14910. 10.1021/acsomega.9b04379. 32637764 PMC7330898

[ref58] Zytner P.; Pal A. K.; Wu F.; Rodriguez-Uribe A.; Mohanty A. K.; Misra M. Morphology and Performance Relationship Studies on Poly(3-hydroxybutyrate-co-3-hydroxyvalerate)/Poly(butylene adipate-co-terephthalate)-Based Biodegradable Blends. ACS Omega. 2023, 8, 1946–1956. 10.1021/acsomega.2c04770. 36687037 PMC9850484

[ref59] Zhang Y.; Zhang H.; Guo W.; Wu C. Effects of different types of polyethylene on the morphology and properties of recycled poly(ethylene terephthalate)/polyethylene compatibilized blends. Polym. Adv. Technol. 2011, 22, 1851–1858. 10.1002/pat.1683.

[ref60] Greene J. P.; 3 - Microstructures of Polymers. In Automotive Plastics and Composites; Greene J. P., Ed.; William Andrew Publishing: New York, 2021; pp 27–37.

[ref61] Kalogeras I. M.; Glass-Transition Phenomena in Polymer Blends. In Encyclopedia of Polymer Blends, Vol. 3: Structure; Isayev A. I., Ed.; Wiley-VCH Verlag GmbH & Co. KGaA: Weinheim, Germany, 2016; pp 1–134.

[ref62] Thirtha V.; Lehman R.; Nosker T. Morphological effects on glass transition behavior in selected immiscible blends of amorphous and semicrystalline polymers. Polymer. 2006, 47, 5392–5401. 10.1016/j.polymer.2006.05.014.

[ref63] Fredi G.; Dorigato A.; Dussin A.; Xanthopoulou E.; Bikiaris D. N.; Botta L.; Fiore V.; Pegoretti A. Compatibilization of Polylactide/Poly(ethylene 2,5-furanoate) (PLA/PEF) Blends for Sustainable and Bioderived Packaging. Molecules. 2022, 27, 6371–6392. 10.3390/molecules27196371. 36234907 PMC9572422

[ref64] Wang B.; Tu Z.; Wu C.; Hu T.; Wang X.; Long S.; Gong X. Effect of Poly(styrene-ran-methyl acrylate) Inclusion on the Compatibility of Polylactide/Polystyrene-b-Polybutadiene-b-Polystyrene Blends Characterized by Morphological, Thermal, Rheological, and Mechanical Measurements. Polymers. 2019, 11, 846–860. 10.3390/polym11050846. 31083318 PMC6572652

[ref65] Collar E. P.; García-Martínez J.-M. A Dynamic Mechanical Analysis on the Compatibilization Effect of Two Different Polymer Waste-Based Compatibilizers in the Fifty/Fifty Polypropylene/Polyamide 6 Blend. Polymers. 2024, 16, 2523–2539. 10.3390/polym16172523. 39274155 PMC11398174

[ref66] Moly K. A.; Bhagawan S. S.; Groeninckx G.; Thomas S. Correlation between the morphology and dynamic mechanical properties of ethylene vinyl acetate/linear low-density polyethylene blends: Effects of the blend ratio and compatibilization. J. Appl. Polym. Sci. 2006, 100, 4526–4538. 10.1002/app.22466.

[ref67] Jiang Z.; Chen H.; Zhao M.; McCarney J.; De Schryver D.; Aplin T.; Sue H.-J. Toughening of linear low-density polyethylene/brominated polystyrene blend by styrene-ethylene/butylene-styrene elastomer. Polymer. 2023, 272, 125859–125867. 10.1016/j.polymer.2023.125859.

[ref68] Ying C.; Zhipeng F.; Min J.; Changjiang S.; Lei X.; Qiang Z.; Guangyuan Z. Preparation and Characterization of Poly(ethylene 2,5-furandicarboxylate)/Poly(butylene succinate) Blends. Chin. J. Appl. Chem. 2015, 32, 1022–1027.

[ref69] Delva L.; Deceur C.; Van Damme N.; Ragaert K. Compatibilization of PET-PE blends for the recycling of multilayer packaging foils. AIP Conf. Proc. 2019, 2055, 030005 10.1063/1.5084815.

[ref70] Abuoudah C. K.; Greish Y. E.; Abu-Jdayil B.; El-said E. M.; Iqbal M. Z. Graphene/polypropylene nanocomposites with improved thermal and mechanical properties. J. Appl. Polym. Sci. 2021, 138, 50024–50036. 10.1002/app.50024.

[ref71] Dedecker K.; Groeninckx G. Interfacial Graft Copolymer Formation during Reactive Melt Blending of Polyamide 6 and Styrene–Maleic Anhydride Copolymers. Macromolecules. 1999, 32, 2472–2479. 10.1021/ma980642v.

