# Supplementary material for: Toughening Brittle Poly(ethylene Furanoate) with Linear Low-Density Polyethylene via Interface Modulation Using Reactive Compatibilizers
Source: ACS Omega. 2025 Feb 4;10(6):5756–69. doi: 10.1021/acsomega.4c09301 (PMC11840768; doi:10.1021/acsomega.4c09301)
Supplement: Supplementary file 1 — ao4c09301_si_001.pdf [file ao4c09301_si_001.pdf]

Toughening Brittle Poly (ethylene Furanoate) with Linear Low-Density Polyethylene via Interface Modulation using Reactive Compatibilizers

Safa Ahmed [1,2], Ruth Cardinaels[2,3], Basim Abu-Jdayil[1], Abdul Munam[4], Muhammad Z. Iqbal[1*]

[1] Chemical and Petroleum Engineering, United Arab Emirates University (UAEU), PO Box 15551, Al Ain, UAE
[2] Department of Chemical Engineering, KU Leuven, Celestijnenlaan 200J, Box 2424, Leuven, 3000, Flanders, Belgium
[3]Department of Mechanical Engineering, Eindhoven University of Technology, P.O. Box 513, Eindhoven, MB 5600, The Netherlands
[4] Department of Biomedical Sciences, University of Niagara Falls, L2E 7J7 Ontario, Canada
*For correspondence: MZ Iqbal; E-mail: mziqbal@uaeu.ac.ae; Tel +971 3 713 5398

Sample Compositions

Table S1 Compositions of PEF and PET blends in weight percentage

Sample	PEF	PET	PE	PE-g-MA	SEBS-g-MA
PEF	100	-	-	-	-
PET	-	100	-	-	-
PEF/PE	50	-	50	-	-
PET/PE	0	50	50	-	-
PEF/PE/PE-g-MA-1.5%	49.25	-	49.25	1.5	-
PEF/PE/PE-g-MA-5%	47.5	-	47.5	5	-
PEF/PE/PE-g-MA-10%	45.0	-	45.0	10	-
PET/PE/PE-g-MA-1.5%	-	49.2525	49.25	1.5	-
PET/PE/PE-g-MA-5%	-	47.5	47.5	5	-
PET/PE/PE-g-MA-10%	-	45.0	45.0	10	-
PEF/PE/SEBS-g-MA-1.5%	49.25	-	49.25	-	1.5
PEF/PE/SEBS-g-MA-5%	47.5	-	47.5	-	1
PEF/PE/SEBS-g-MA-10%	45.0	-	45.0	-	10
PET/PE/SEBS-g-MA-1.5%	-	49.2525	49.25	-	1.5
PET/PE/SEBS-g-MA-5%	-	47.5	47.5	-	5
PET/PE/SEBS-g-MA-10%	-	45.0	45.0	-	10

Prediction of Polymer (Im)miscibility

Owing to their low entropy of mixing, polymers are typically immiscible with each other, and immiscibility may result in significantly improved properties exceeding the individual properties of the blended components. Thus, identifying and understanding the extent of miscibility/immiscibility of the blend components gives important insights in the anticipated resultant properties. The solubility parameter (δ) is a very good indicator of immiscibility/miscibility of polymers. According to Van Krevelen [1], miscibility of two substances is possible if the difference between their solubility parameters ($\Delta\delta$) is small, for instance $\Delta\delta < 5$ MPa$^{0.5}$.

The solubility parameter (δ) is defined by the cohesive energy (E_{coh}) through the correlation[1]:

$$\delta = \left(\frac{E_{coh}}{V}\right)^{0.5} \quad (1)$$

where V is the molar volume. E_{coh} comprises of dispersive (E_d), polar (E_p), and hydrogen bonding (E_h) interactions as follows [1]:

$$E_{coh} = E_d + E_p + E_h \quad (2)$$

Similarly, the overall δ of a polymer contains three components [1]:

$$\delta^2 = \delta_d^2 + \delta_p^2 + \delta_h^2 \quad (3)$$

where δ_d, δ_p, and δ_h are the dispersive, polar, and hydrogen bonding component of the solubility parameter, respectively.

The miscibility/immiscibility of a binary polymer system is evaluated based on the solubility difference of the individual components calculated as follows [1]:

$$\left(\Delta\delta^2\right) = [\delta_2 - \delta_1]^2 = [\delta_{d,2} - \delta_{d,1}]^2 + [\delta_{p,2} - \delta_{p,1}]^2 + [\delta_{h,2} - \delta_{h,1}]^2 \quad (4)$$

The group contribution method is a viable approach to estimate δ, and several methods are reported for δ calculation[1]. The methods of Hoftyzer and Van Krevelen (1970) and Hoy's system (1985) allow a systematic estimation of δ_d, δ_p, and δ_h individually, whereas the approaches of Small, Hoy and Van Krevelen determine only the overall solubility parameter [1-5]. In this study, the Hoftyzer and Van Krevelen method was selected to determine the solubility parameters of PEF, PET, and PE. Accordingly, the individual solubility parameters were calculated as follows [1]:

$$\delta_d = \frac{\sum F_{d_i}}{V} \qquad \delta_p = \frac{\sqrt{\sum (F_{p_i})^2}}{V} \qquad \delta_h = \sqrt{\frac{\sum E_{h_i}}{V}} \quad (5)$$

where V is the total molar volume determined as the summation of the molar volumes of the functional groups, calculated here using Hoy's estimations [1], F_{di} (in (MJ.m^3)$^{0.5}$/mole) and F_{pi} (in (MJ.m^3)$^{0.5}$/mole) are the group contributions to the dispersive and polar molar attraction, respectively, whereas E_{hi} (in J/mole) is the group contribution to the hydrogen bonding energy. Calculated solubilities of PEF, PET, PE are exhibited in Table S1.

While the Hoftyzer and Van Krevelen method allows for determining the contribution of the benzene ring to the total solubility parameter of PET directly, it does not provide a direct estimation of the furan ring contribution. Therefore, the furan ring was split into 5 functional groups and their contributions were estimated individually and then added to reflect the ring contribution. For the total molar volumes, data provided by Hoy[1] do not encompass the total

molar volumes of rings. Therefore, the molar volumes of the rings for both PEF and PET were determined based on their functional groups (Table S3).

Table 1 contains the overall molar volumes and calculated δ parameters of the repeat units of PET, PEF and PE. The δ of PET and PEF are in a good agreement with the data reported by Poulopoulou et al [6], who used a similar method for δ estimation. Besides, the δ of PET and PE exhibited insignificant deviations from the δ calculated by Hoftyzer and Van Krevelen [1] (Table S4). These small differences might be attributed to the different methods for determining the molar volume, as we adopted Hoy's values, while Hoftyzer and Van Krevelen [1] considered experimental data.

Table S2 Polymer molar volumes (V) and solubility parameters (δ)

Polymer	V cm^3/mol	Δδ MPa$^{0.5}$	δ_d MPa$^{0.5}$	δ_p MPa$^{0.5}$	δ_h MPa$^{0.5}$
PET	145.58	21.41	17.79	6.77	9.81
PEF	125.67	22.34	17.11	8.42	11.63
PE	32.2	17.36	17.36	0	0

PEF and PET have similarities in their chemical structures (see Scheme. 1), both comprise ethylene functional groups (-CH_2-CH_2-) and 2 carboxylic groups (-COO-). A significant difference between the two polymers is that PET contains a 6-C aromatic ring compared to the heterogenous furan ring in PEF that comprises of 4 carbons and one oxygen atom. This dissimilarity leads to polar attraction in the furan ring attributed to the oxygen, whereas very low polar attractions were found in PET's benzenic ring. On the other hand, the dispersion molar attraction is higher in the benzenic ring compared to the furan ring, and consequently, δ is not anticipated to differ significantly between the two polyesters.

Calculation of Individual Solubility Parameters

Determination of individual solubility parameters $\delta_d, \delta_p, \delta_h$, were determined applying the Hoftyzer and Van Krevelen (H-V) method as follows [1]:

$$\delta_d = \frac{\sum F_{d_i}}{V} \qquad \delta_p = \frac{\sqrt{\sum (F_{p_i})^2}}{V} \qquad \delta_h = \sqrt{\frac{\sum E_{h_i}}{V}} \tag{6}$$

where F_{di} ((MJ.m^3)$^{0.5}$/mole) and F_{pi} ((MJ.m^3)$^{0.5}$/mole) are group contributions to the dispersive and polar molar attraction, respectively, whereas E_{hi} (J/mole) is the group contribution to the hydrogen bonding forces, and V is the total molar volume.

The group contribution to the different parameters specified in H-V method for each functional group of the repeat unit of PEF, PET, PE were determined as illustrated in Table S3.

Table S3 Group contributions to the parameters of (H-V) method

Polym.	Chemical structure (repeat unit)	Group	F_{di} $(MJ.m^3)^{1/2}/mol$	F_{pi} $(MJ.m^3)^{1/2}/mol$	E_{hi} J/mol	V_i (Hoy) cm^3/mol	n_i*
**PEF**	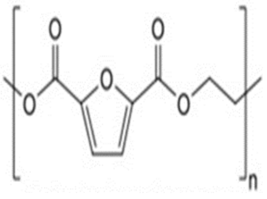	-CH2-	270.00	0.00	0.00	15.55	2
		-COO-	390.00	490.00	7000.00	23.70	2
		-CH=	200.00	0.00	0.00	13.18	2
		>C=	70.00	0.00	0.00	7.18	2
		-O-	100.00	400.00	3000.00	6.45	1
		Ring	190.00	0.00	0.00	0.00	1
**PET**	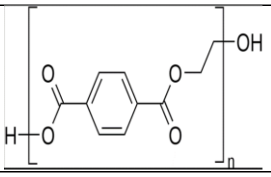	-CH2-	270.0	0.0	0.0	15.55	2
		-COO-	390.0	490.0	7000.0	23.70	2
		Benzene	1270.0	110.0	0.0	13.18	1
**PE**	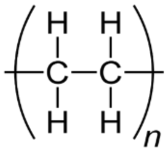	CH2--	270.00	0.00	0.00	15.55	2

* Number of the functional group in the repeat units

Table S4 Comparison of δ for PEF, PET, PE from different references

Method	Material	V (cm^3/mol)	δ $(MPa)^{0.5}$	δ_d $(MPa)^{0.5}$	δ_p $(MPa)^{0.5}$	δ_h $(MPa)^{0.5}$	Ref.
**H-V**	PEF	125.67	22.34	17.11	8.42	11.63	Calculated
	PET	145.58	21.41	17.79	6.77	9.81	
	PE	32.2	17.36	17.36	0	0	
**H-V**	PEF	125.7	22.5	-	-	-	[6]
	PET	144.2	22.0	-	-	-	
**H-V**	PET	143.2	20.5	-	-	-	[1]
	PE	32.9	16	-	-	-	

Free Surface Energy Measurement and Interfacial Tension Calculations

The surface free energy of pure PEF was determined applying Owens-Wendt method [7]. This method allows calculating the polar, and dispersion elements of the free surface energy as well (equations (7), and (8)).

The contact angle was measured using two different liquids; water, and glycerol, with the known surface tensions listed in Table S5 [8].

$$\left(\gamma_s^d \gamma_{l1}^d\right)^{0.5} + \left(\gamma_s^p \gamma_{l1}^p\right)^{0.5} = 0.5\gamma_{l1}(1+\cos\theta_1) \quad (7)$$

$$\left(\gamma_s^d \gamma_{l2}^d\right)^{0.5} + \left(\gamma_s^p \gamma_{l2}^p\right)^{0.5} = 0.5\gamma_{l2}(1+\cos\theta_2) \quad (8)$$

Where γ_s , γ_s^d , and γ_s^p are the surface tension, dispersion, and polar elements of the surface γ_{li} , γ_{li}^d , and γ_{li}^p are the surface tension, dispersion, and polar elements of the surface tension of liquid i, respectively. θ_1 and θ_2 are the contact angles between droplets of liquid's 1, and 2 and PEF surface.

Table S5 Surface tension of water, and glycerol

The

**Liquid**	γ_l	γ_l^d	γ_l^p
**Water (W)**	72.8	21.8	51.0
**Glycerol**	64.0	34.0	30.0

average measured contact angles θ_1 and θ_2 at 20°C were found to be 68.5°, and 51°, respectively. To calculate the free surface energy of the materials at processing temperatures (270°C for PET/PE blends, and 240°C for PEF/PE blends), the following formal was applied:

$$\gamma_T = \gamma_0 + \kappa(T - 20) \quad (9)$$

where γ_T is the free surface energy at a specific temperature T , γ_0 is the free surface energy at 20°C, κ is the material's temperature coefficient. It is important to notice that κ for PEF is not reported in literature, hence it was considered here as the typical value of PET's.

The free surface energy of PEF, PET, PE at 20°C, and processing temperatures are listed in Table S6.

Table S6 Free Surface energy of blend components

Polymer	Free Surface Energy at 20 ˚C mN/m			Temp. coff.	Free Surface Tension at processing temp. mN/m			
	Total	Dispersive Energy	Polar Energy		Total	Dispersive	Polar	Temp. °C.
PEF[II]	47.81	39.72	8.09	-0.065	33.51	27.84	5.67	240
PET[I]	44.6	35.6	9	-0.065	28.48	22.6	5.7	270
PE[I]	35.7	35.7	0	-0.057	23.60	23.60	0	240
					21.45	21.45	0	270
PE-g-MA[III]	43.33	37.6	1.2	-0.057	30.8	29.9	0.9	240
					29.1	28.2	0.87	270
SEBS-g-MA[III]	33.7	33.1	0.57	-0.045	23.76	23.35	0.41	240
					22.4	22.0	0.38	270

[I9] [II] Experimental [III10]

Morphologies of Compatibilized Blends

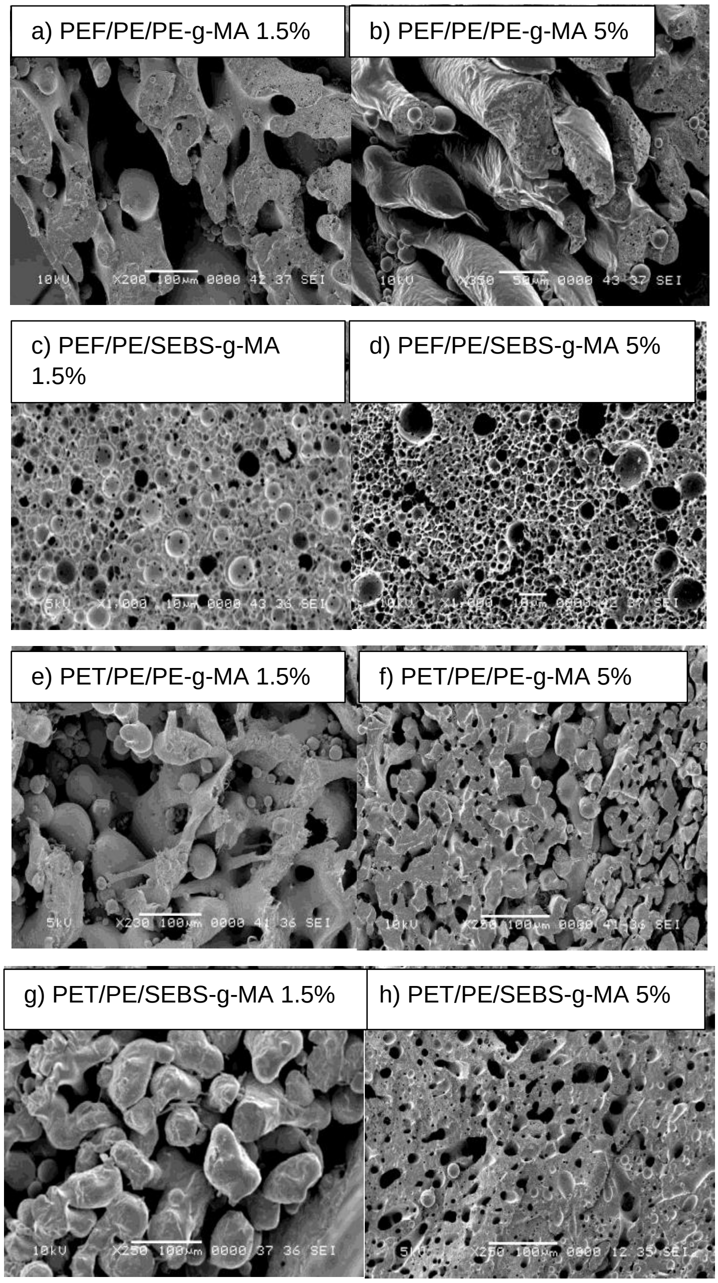

Figure S1 Morphology of PEF/PE and PET/PE 50/50 blends with various concentrations of PE-g-MA or SEBS-g-MA

Complex Viscosity

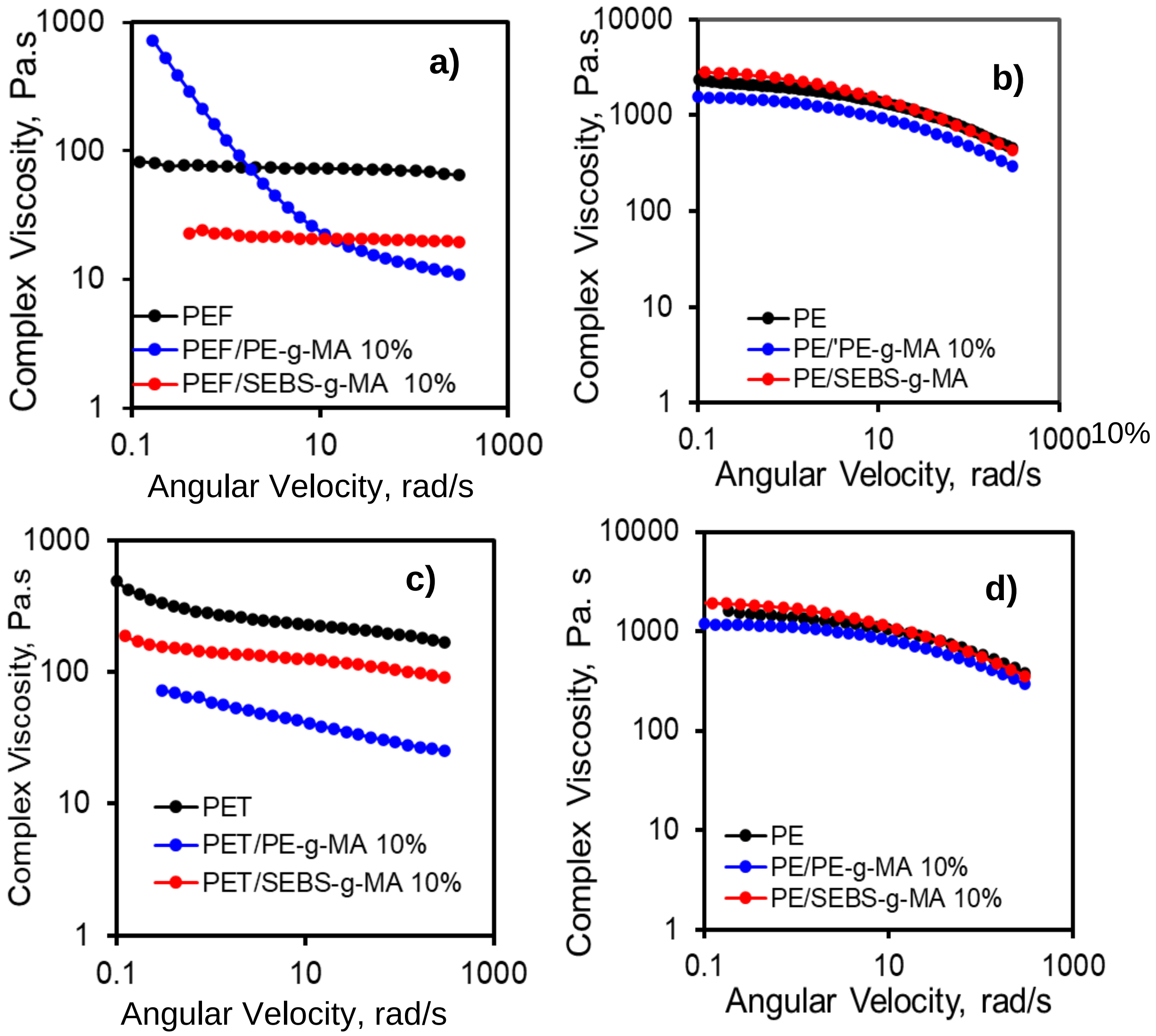

Figure S2 Complex viscosity (frequency sweep test) of a) PEF/compatibilizers, b) PE/Compatibilizers @ 240 °C, c) PET/compatibilizers, d) PE/Compatibilizers @ 270 °C

Shear Rate Calculation

To calculate the average shear rate ($\dot{\gamma}$) at the compounding conditions, the following formula [11] was applied:

$$\dot{\gamma} = \frac{2\pi NR}{60h} \tag{10}$$

where N is the rotating speed (rpm), R is the screw radius, h is the channel depth. For the current study, N is 50 rpm, the average R, and h are 9.5, and 4.5 mm, respectively. Consequently, $\dot{\gamma}$ was determined as 5.53 s^{-1}

Cocontinuity Models

Jordhamo or Miles-Zurek Model: $$\frac{\phi_1}{\phi_2} = \frac{\eta_1(\dot{\gamma})}{\eta_2(\dot{\gamma})}$$ (11) [12]

Ho et al. Model: $$\frac{\phi_1}{\phi_2} = 1.22\left(\frac{\eta_1(\dot{\gamma})}{\eta_2(\dot{\gamma})}\right)^{0.29}$$ (12) [13]

Metelkin-Blekht Model: $$\frac{\phi_1}{\phi_2} = \frac{\eta_1(\dot{\gamma})}{\eta_2(\dot{\gamma})} F\left(\frac{\eta_1(\dot{\gamma})}{\eta_2(\dot{\gamma})}\right)$$

$$F\left(\frac{\eta_1(\dot{\gamma})}{\eta_2(\dot{\gamma})}\right) = 1 + 2.25\log\left(\frac{\eta_1(\dot{\gamma})}{\eta_2(\dot{\gamma})}\right) + 1.81\left[\log\left(\frac{\eta_1(\dot{\gamma})}{\eta_2(\dot{\gamma})}\right)\right]^2$$ (13) [14]

Utracki Model: $$\phi_2 = \frac{1}{2}\left(1 - \frac{\log\left(\frac{\eta_1(\dot{\gamma})}{\eta_2(\dot{\gamma})}\right)}{[\eta]}\right)$$ (14) [15]

where $\emptyset_1$ is volume fraction of PEF(PET) in their blends, $\emptyset_2$ is volume fraction of PE in the blends, η_1 is viscosity of PEF/PET (uncompatibilized/compatibilized) at the mixing shear rate, η_2 is the viscosity of PE (uncompatibilized/compatibilized) at the mixing shear rate.

The models were fitted for the compositions of PEF/PE, (PEF /PE-g-MA 10%) /(PE/PE-g-MA 10%), (PEF /SEBS-g-MA 10%)/(PE/SEBS-g-MA 10%PET/PE), (PET/PE-g-MA 10%)

/(PE/PE-g-MA 10%), (PET/SEBS-g-MA10%) /(PE/SEBS-g-MA 10%). Hereby, it is assumed that the compatibilizer is present with 5 wt.% in both phases.

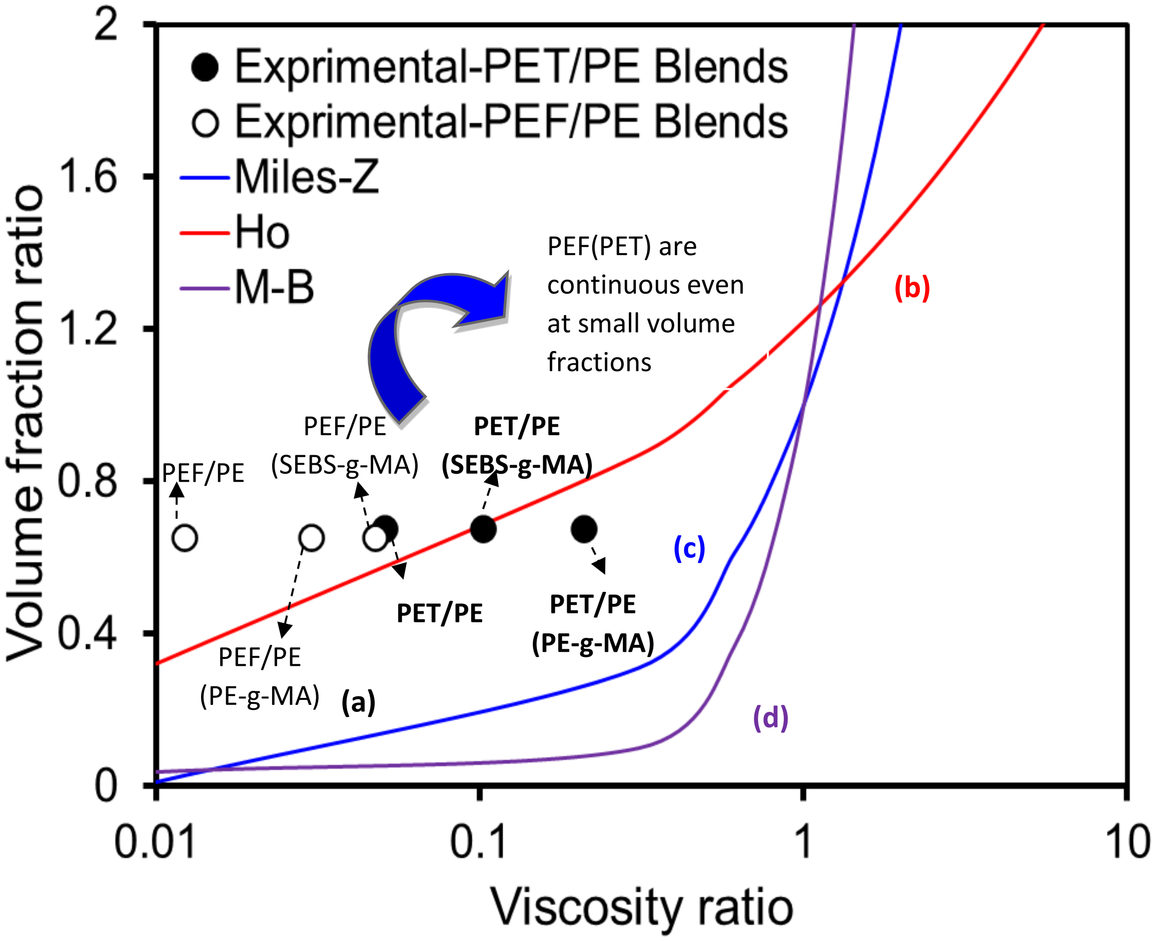

Figure S3 Comparing experimental viscosity ratio data with models: a) Experimental data including: PEF/PE, (PEF/PE-g-MA 10%)/(PE/PE-g-MA 10%),(PEF/SEBS-g-MA 10%)/(PE/SEBS-g-MA 10%), PET/PE, (PET/PE-g-MA 10%)/(PE/PE-g-MA 10%),(PET/SEBS-g-MA 10%)/(PE/SEBS-g-MA 10%), b) Ho et al. c) Miles-Zurek, d) Metelekin-Blekht

FTIR Results

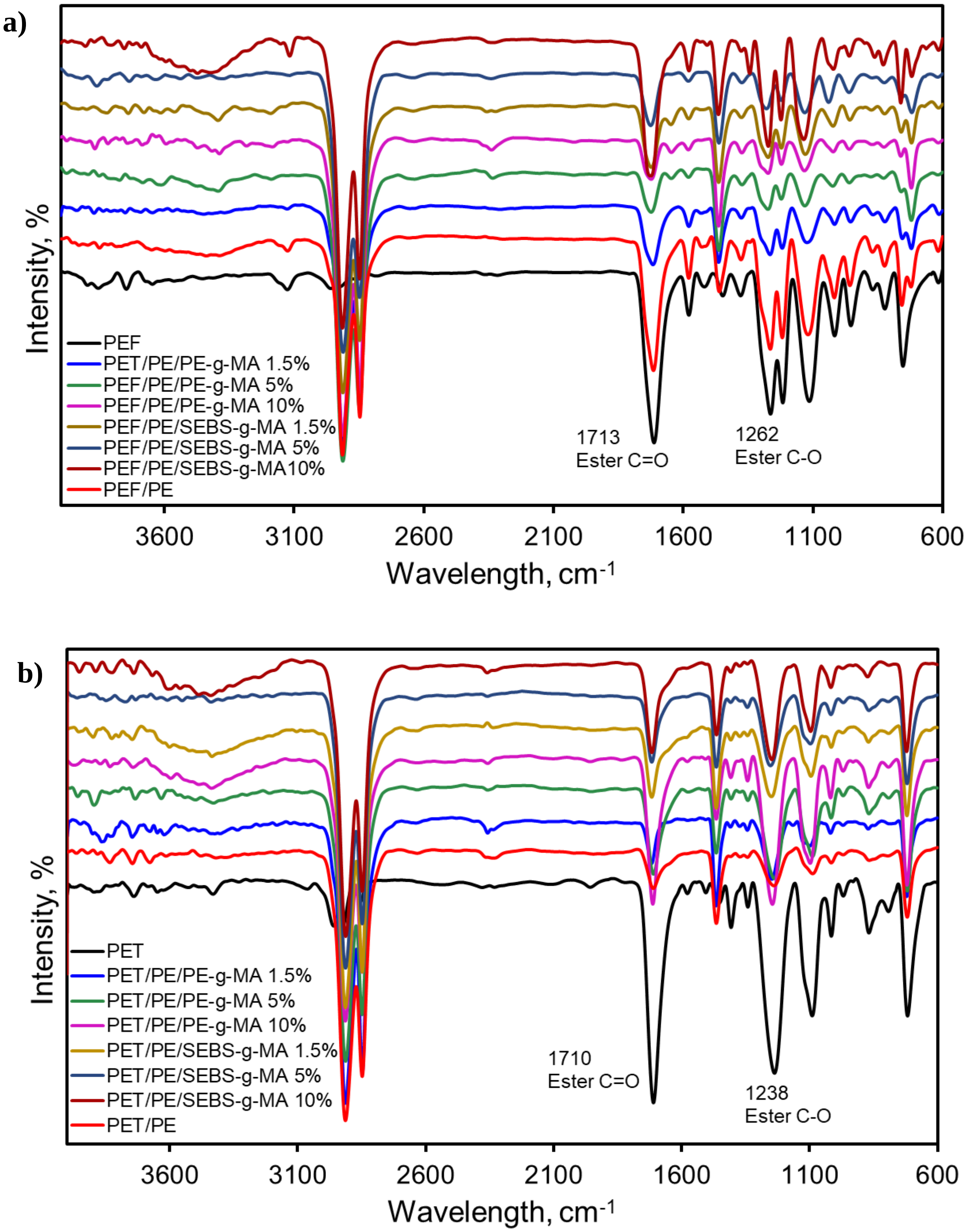

Figure S4 FTIR spectrum of a) PEF/PE blends, b) PET/PE blends

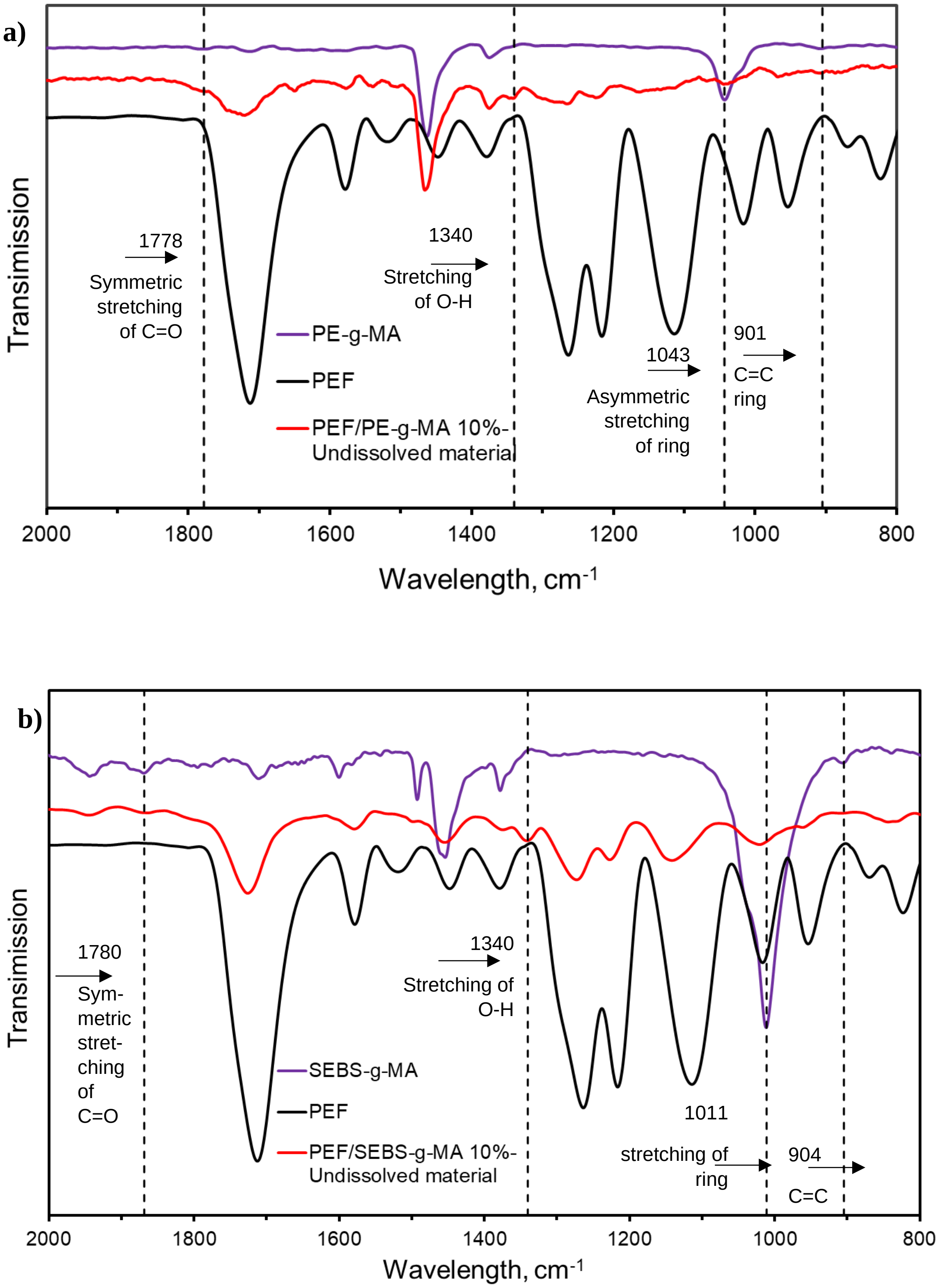

a)
Transimission
1778
Symmetric stretching of C=O
1340
Stretching of O-H
PE-g-MA
PEF
PEF/PE-g-MA 10%-Undissolved material
1043
Asymmetric stretching of ring
901
C=C ring
2000
1800
1600
1400
1200
1000
800
Wavelength, cm-1
b)
Transimission
1780
Sym-metric stret-ching of C=O
1340
Stretching of O-H
SEBS-g-MA
PEF
PEF/SEBS-g-MA 10%-Undissolved material
1011
stretching of ring
904
C=C
2000
1800
1600
1400
1200
1000
800
Wavelength, cm-1

c)

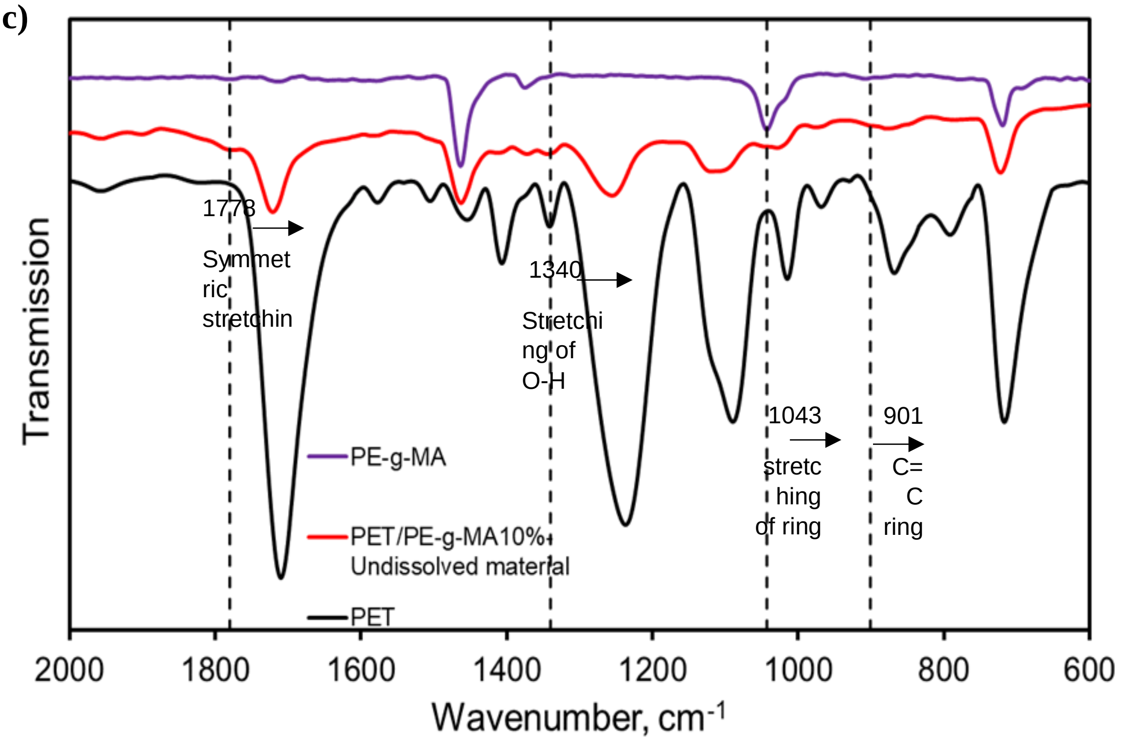

d)

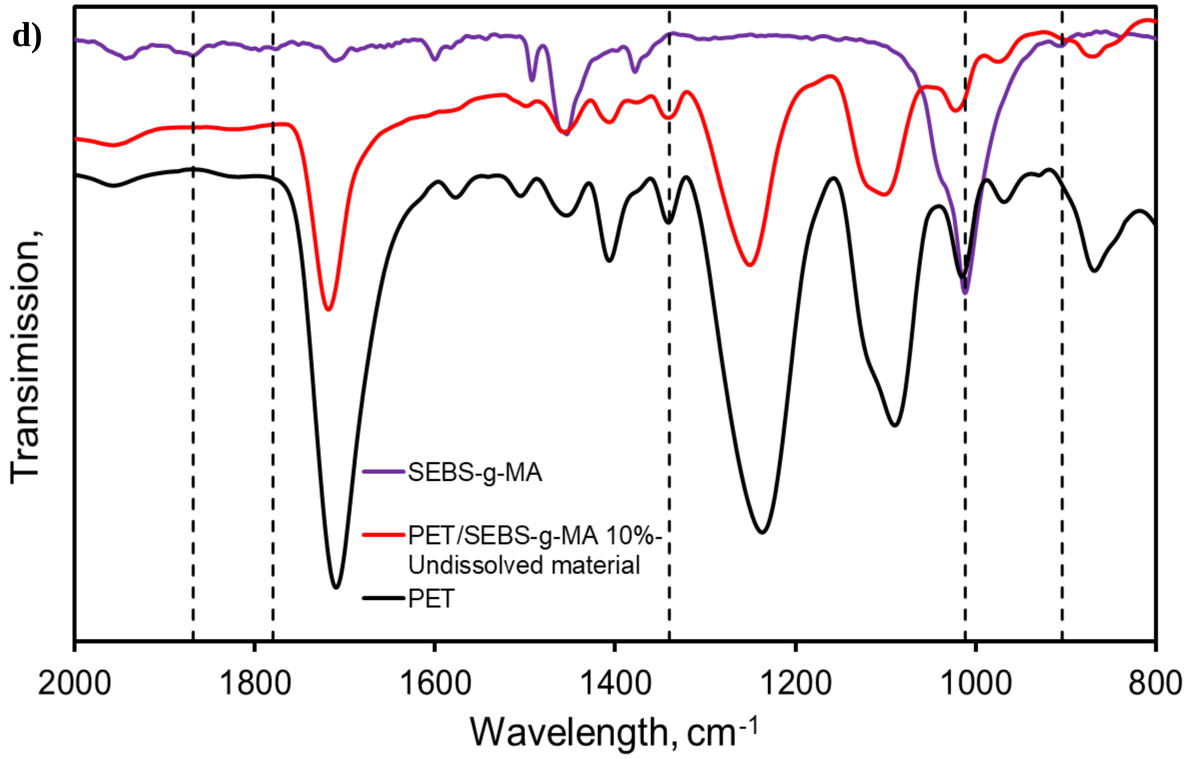

Figure S5 FTIR spectrum of a) PEF/PE-g-MA blend, b) PEF/SEBS-g-MA blend, c) PET/PE-g-MA blend d) PET/SEBS-g-MA blend

Chemical Inteactions

a

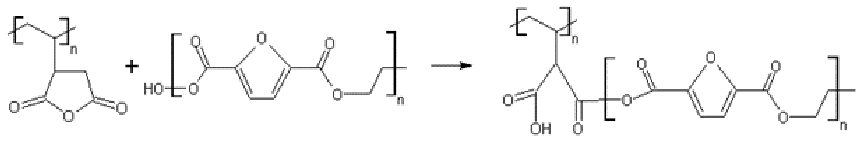

b

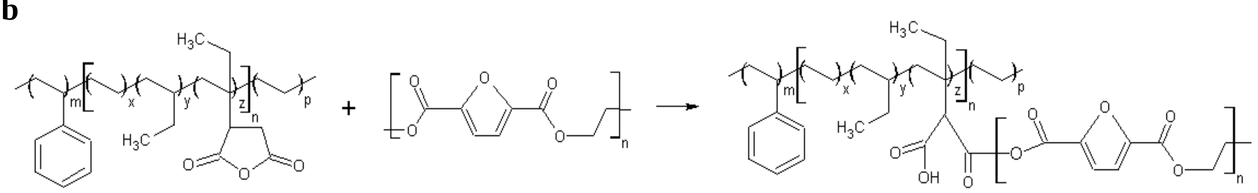

c

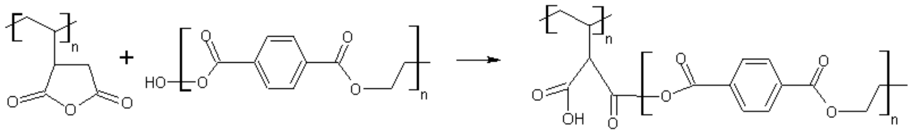

d

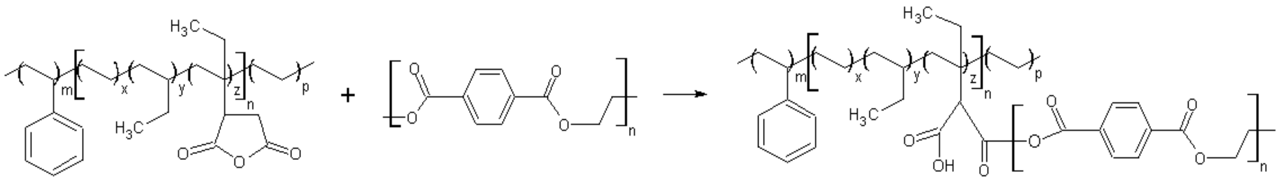

Scheme. S1 Interaction schemes of a) PEF/PE-g-MAb) PEF/SEBS-g-MA, c) PET/PE-g-MA, d) PET/SEBS-g-MA

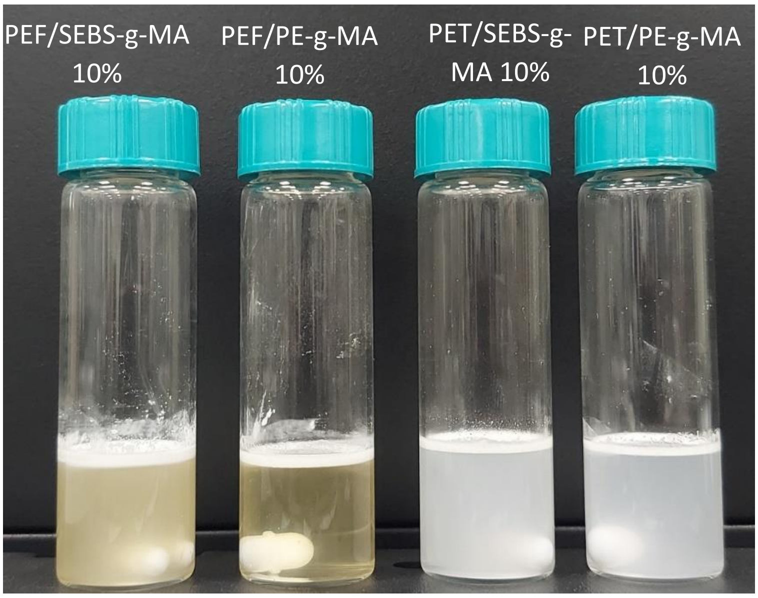

Figure S6 solutions of Polyester/compatibilizer

GPC Results

a)

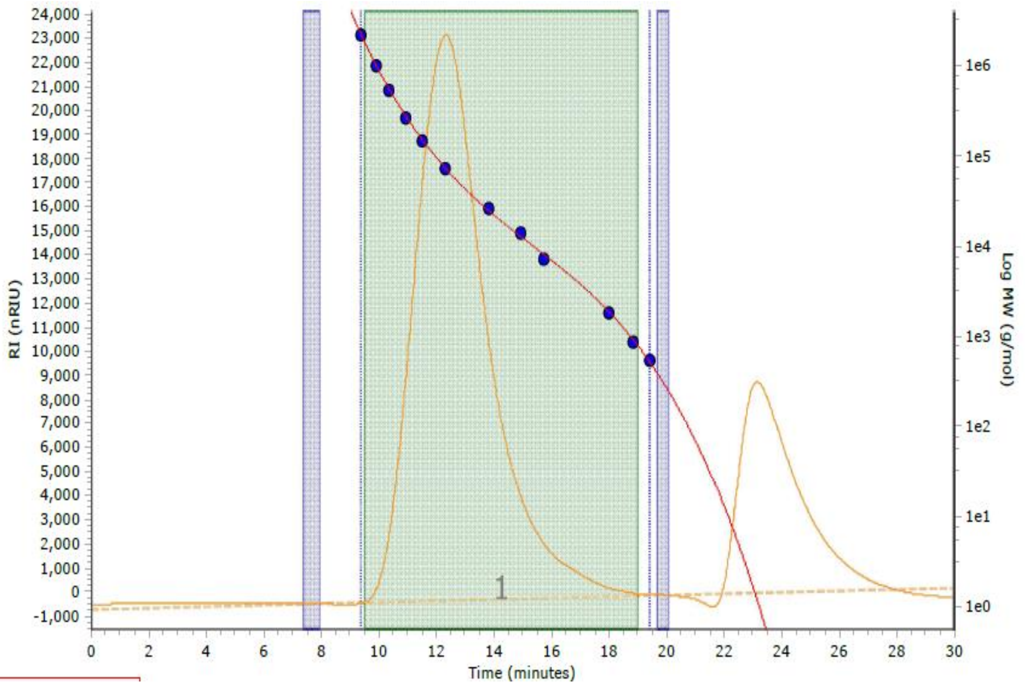

b)

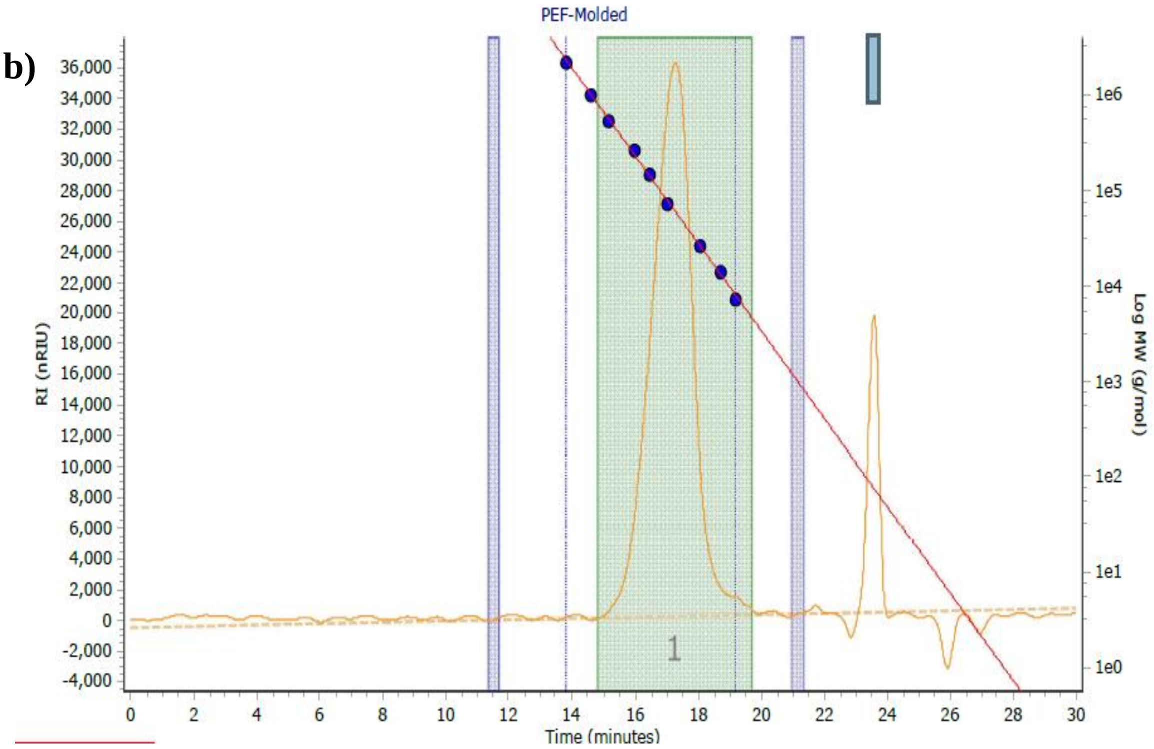

C)

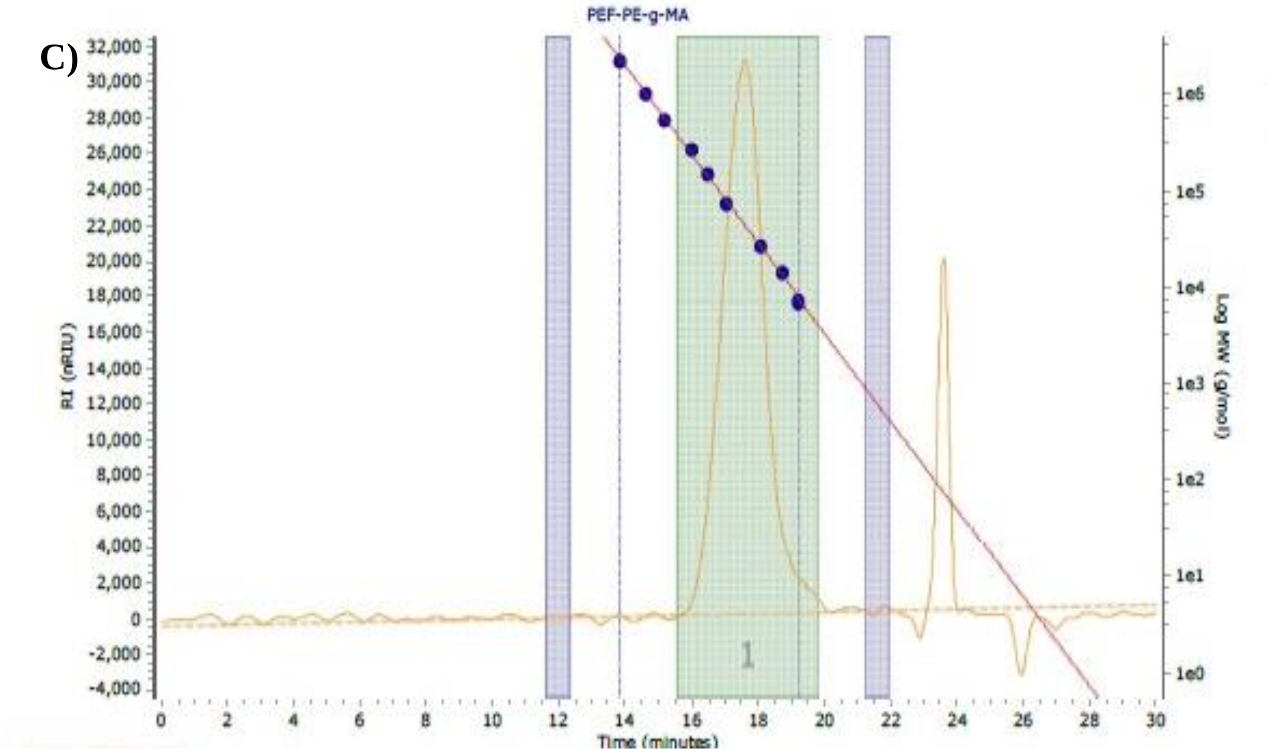

Figure S7 Chromatogram of a) as-received PEF, and b) molded PEF C) PEF/PE-g-MA 10 wt%

Table S7 Molecular weights of materials

Material	Mn	Mw	PD
**PEF (as-received)**	31018	93868	3.03
**PEF (moulded)**	51406	85311	1.66
**PEF/PE-g-MA**	33594	53770	1.60

XRD Results

Figure S8 depicts the XRD patterns of the pure PEF and PE and the PEF/PE blends. Neat PEF showed only a broad diffraction peak, indicating a total amorphous structure [16]. On the other hand, diffraction peaks of PE appeared at 21.9° and 24.2° refer to the (110) and (200) reflections of the orthorhombic crystal system known for polyethylene[17]. The diffraction peaks were retained with a tiny shift in the angles in all blends indicating that the crystal structure of the pure PE was not altered by blending. Yet, peak's intensity decreased noticeably compared to the neat PE, which is anticipated due to the lower fraction of PE existing in the blends. Likewise, PE crystalline peaks that existed in all PET/PE blends showed a slight shift in 2θ and less intensity. PET triclinic structure was confirmed by the peaks at 2θ degrees of 16.4, 17.8, , 21.9, 23 and 26.5° which characterizes (011), (010), (110), (111) to and (1 0 0) planes, respectively[18]. Similar to the PEF/PE blends, the chemical structures of the pure component were preserved in

the blends, accompanied with a decrease in the peak's intensity due to the lower fractions of the components present in the blends.

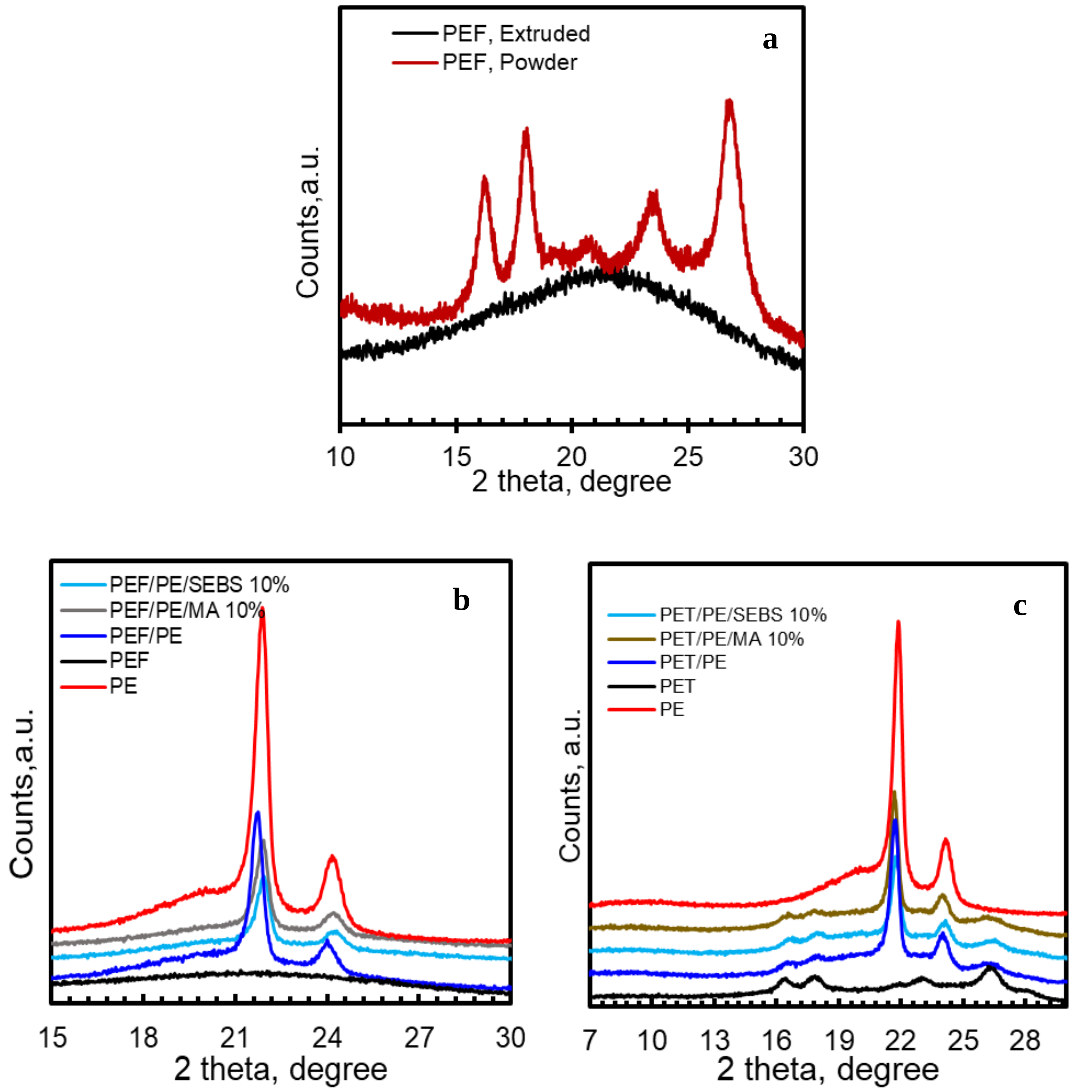

Figure S8 XRD patterns of a) PEF (extruded), and PEF (powder) b) PEF/PE Blends, c) PET/PE Blends

Effects of the Processing Conditions on PEF Properties

Before conducting a thorough thermal analysis on PEF, we compared as-received and extrusion-molded (molded) PEF samples to see any significant modifications in PEF inflicted due to processing. The difference noted in the FTIR spectra, as seen in Figure 4b, between the as-received and molded PEF is the absence of a peak at 1340 cm^{-1} in the molded PEF, which is existent in the as-received PEF. A previous FTIR analysis on the PEF structure conducted by Arouja et al. [19] attributed the peaks at 1340 cm^{-1} and 1477 cm^{-1} to the crystalline structure of PEF, stemming from trans-ethylene glycol (EG) whereas the same group vibrates at 1370 cm^{-1} and 1455 cm^{-1} for gauche-EG showing amorphous PEF. Also, this research group reported that the furan ring arising from FDCA (sync-FDCA) shows crystalline vibrations at 1577 cm^{-1} and 609 cm^{-1} whereas anti-FDCA vibrations at 1582 cm^{-1} and 618 cm^{-1} indicate amorphous PEF. In the current study, these findings suggest the crystalline structure of the as-received PEF,

contrasting with the more amorphous structure in the molded PEF. This observation is supported by the XRD patterns (Fig. S8a). The powdered as-received sample exhibited crystalline peaks at 16.2°, 18°, 19.4°, 20.7°, 23.5°, and 26.8° indicating a triclinic structure similar to that of PET[18]. Conversely, the extruded and molded sample exhibited a total amorphous structure.

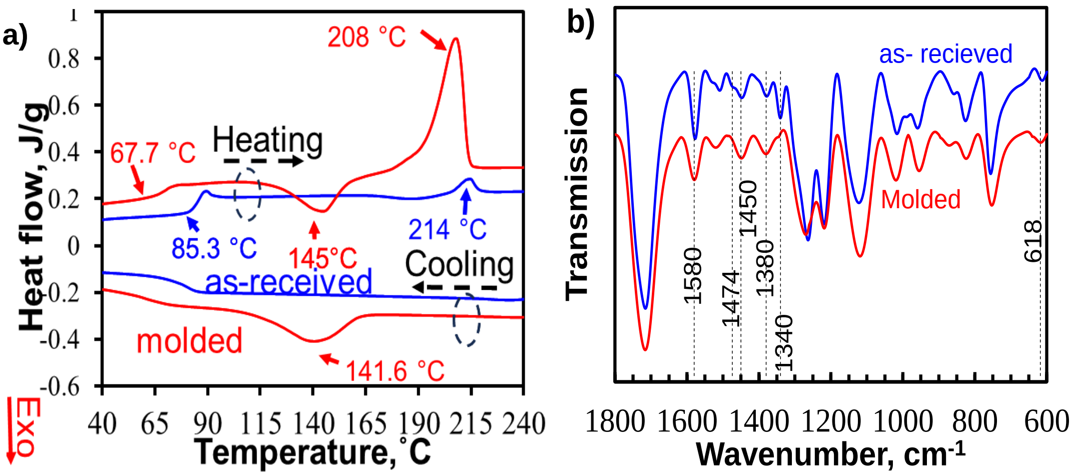

Figure S9 Comparison between as-received and molded PEF **a)** second heating and first cooling cycles of as-received and molded PEF, **b)** FTIR spectrum of as-received and molded PEF

However, some studies reported an amorphous structure for the as-synthesized PEF[16, 20], and the crystalline structure was only observed in PEF after induced crystallization[19, 20]. On the other hand, DSC observations (Figure S9a) revealed the rapid crystallization behavior within the extruded PEF at 141.6°C upon cooling as well as cold crystallization at 145 °C during heating, in contrast to its absence in the as-received form, which is in agreement with a previous work [21]. Furthermore, the glass transition temperature (T_g) decreased by ~15°C after molding, together with a decrease in melting peak temperature from 214°C for as-received PEF to 208°C for molded PEF. These variations can be ascribed to the reduction in the molecular weight of the polyester backbone due to extrusion and molding as elaborated further below, enhancing its segmental mobility and consequently leading to a reduction in T_g while enhancing the degree of molecular order which may promote the tendency for crystallization.For assessing the effects of processing, the molecular weights of both samples were measured. The as-received PEF showed Mw = 94 kg/mol and Mn = 31 kg/mol which decreased to Mw = 85 kg/mol while Mn increased to 51 kg/mol for molded PEF. The corresponding PDI values were 3.2, and 1.7, respectively. The decrease in Mw along with the decrease in PDI detected in molded PEF is evidence of long chain scission. On the other hand, this reduction in Mw may also explain the increase in crystallinity from 2.4% in unprocessed PEF to 20.6% in molded PEF [22]. Generally, the structural change during processing of PEF is noteworthy towards practical implementation of PEF applications. Nevertheless, a detailed study on the effects of processing conditions on the PEF structure is beyond the scope of the present work.

Thermal Properties

Table S8 Thermal Properties of PEF, PET blends

Sample ID	Sample	T_m (˚C)		T_c (˚C)		T_g
		PEF /PET	PE	PEF/ PET	PE	PEF/ PET
1	PEF (molded)	207.9	-	-		69.9
2	PEF/PE	208.0	119.7	-	105.0	69.7
3	PEF/PE/PE-g-MA 1.5%	209.9	119.3	-	105.2	71.1
4	PEF/PE/PE-g-MA 5%	213.3	120.8	-	105.3	81.3
5	PEF/PE/PE-g-MA 10%	210.4	119.5	-	104.7	76.3
6	PEF/PE/SEBS-g-MA 1.5%	206.5	121.1	-	105.6	67.7
7	PEF/PE/SEBS-g-MA 5%	209.4	120.0	-	105.8	70.6
8	PEF/PE/SEBS-g-MA 10%	207.2	121.5	-	105.9	68.6
9	PE (molded)	-	120.0	-	106.8	-
10	PET (molded)	250.6	-	215.5	-	-
11	PET/PE	252.5	121.1	213.3	103.0	60.8
15	PET/PE/PE-g-MA 1.5%	251.6	120.3	210.5	104.	60.9
16	PET/PE/PE-g-MA 5%	252.2	120.0	207.7	103.4	63.5
17	PET/PE/PE-g-MA 10%	251.2	117.8	214.1	103.6	59.9
12	PET/PE/SEBS-g-MA 1.5%	252.1	120.4	212.4	105.0	60.8
13	PET/PE/SEBS-g-MA 5%	251.3	120.3	201.9	103.1	61.5
14	PET/PE/SEBS-g-MA 10%	251.1	119.3	210.4	105.4	64.0

Interaction Parameter Calculations

Polymer-polymer interaction parameter (χ12) can be calculated using Nishi-Wang equation based on Flory-Huggins theory [23] as follows:

$$\frac{1}{T^o_{m,b}} - \frac{1}{T^o_m} = -\frac{RV_2}{\Delta H_2 V_1} \chi 12 \phi_1^2 \qquad (15)$$

Where T°_{m} and $T^{\circ}_{m,b}$ are equilibrium melting points of neat and blended crystalline component of the blend (PE in this study), respectively; R is the universal gas constant (8.314 J $mol^{-1}K^{-1}$); V_1, V_2 are molar volumes of repeat units of PET or PEF and PE, respectively; ΔH_2 is heat of fusion for 100% crystalline PE (279 J/mol), ϕ_1 is the volume fraction of the PET or PEF in the polymer blend.

If significant intermolecular interactions occur between the blend components, the equilibrium melting point of the crystallizable polymer in the mixture should be lower than that of the pure crystalline polymer. This phenomenon, known as equilibrium melting point depression, is expected when the χ12 is negative[24]. The extent of this depression is primarily influenced by the strength of the interactions and the concentration of the crystalline component in the blend.

Conversely, if χ12 is positive, an equilibrium melting point elevation may be anticipated. However, in a polymer mixture where χ12 is positive, the system tends to become incompatible with phase-separated molecules. In such cases, the thermodynamic assumptions underlying Equation (15) may be deemed invalid, and crystallization and subsequent melting behavior may resemble that observed in the pure polymer [23].

Given the demonstrated total incompatibility of the polyester/PE blends, it is evident that the Nishi-Wang equation may not be suitable for this system. Nevertheless, by introducing compatibilization, a degree of miscibility between the components becomes feasible. Therefore, χ12 was determined by applying equation (15) to assess the miscibility of the compatibilized blends.

To determine the equilibrium melting temperatures, samples of neat PE, compatibilized and un-compatibilized blends of PEF/PE and PET/PE were isothermally crystallized at 110, 113, 116, and 119˚C for suitable crystallization times, and the apparent melting points of PE were determined, sequentially, Hoffman-Weeks graph for pure PE and blends were plotted, and equilibrium melting points of neat PE and PE in blends were determined from the plots **(**Figure S10). It was found that the equilibrium melting point of PE (131.77˚C) in the pure polymer which slightly increased in the uncompatibilized blends, while surprisingly increased by 0.27 to 2.3˚C in the compatibilized blends. One possible explanation for this elevation is the phenomenon of crystal perfection. In blends containing high concentrations of "impurities," which may constitute the other phase or a portion of the compatibilizer, the presence of high molecular weight impurities can potentially enhance crystalline perfection. Consequently, an increase in blend melting point can be anticipated with increasing levels of crystal perfection [23].

Applying the determined equilibrium melting points in (Flory; Nishi and Wang) equation to estimate the interaction in uncompatibilized and compatibilized PEF/PE and PET/PE blends yielded values ranged from 0.009 to 0.106. The positive value indicates immiscibility of the systems, as a consequence of the lacking melting points depression, as discussed previously. For uncompatibilized systems, this finding is in agreement with the solubility parameters prediction. However, for the compatibilized blends, these results are not conclusive, as the lack of depression may stem from other thermodynamic factors.

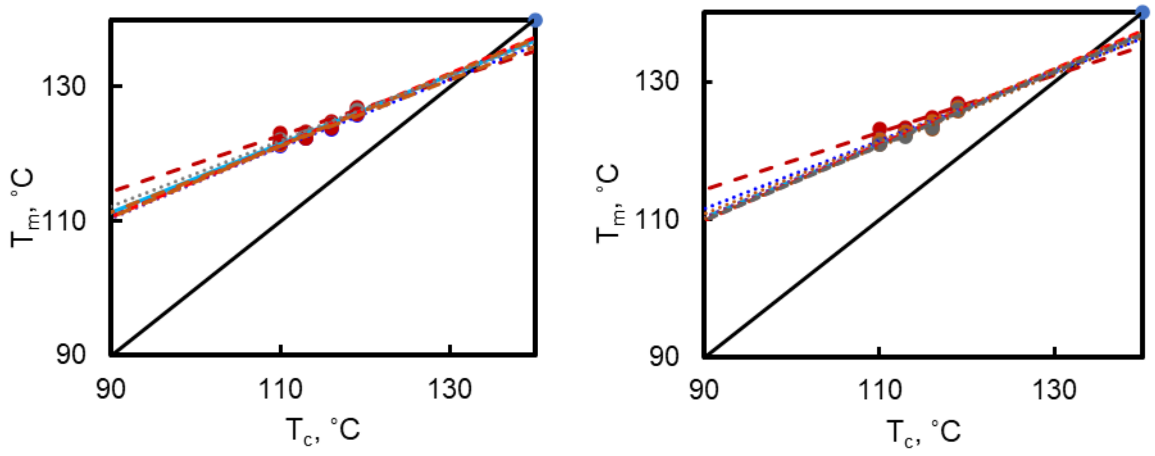

Figure S10 Hoffman-Weeks plots of a) PEF/PE blends, **b)** PET/PE blends

TGA Results

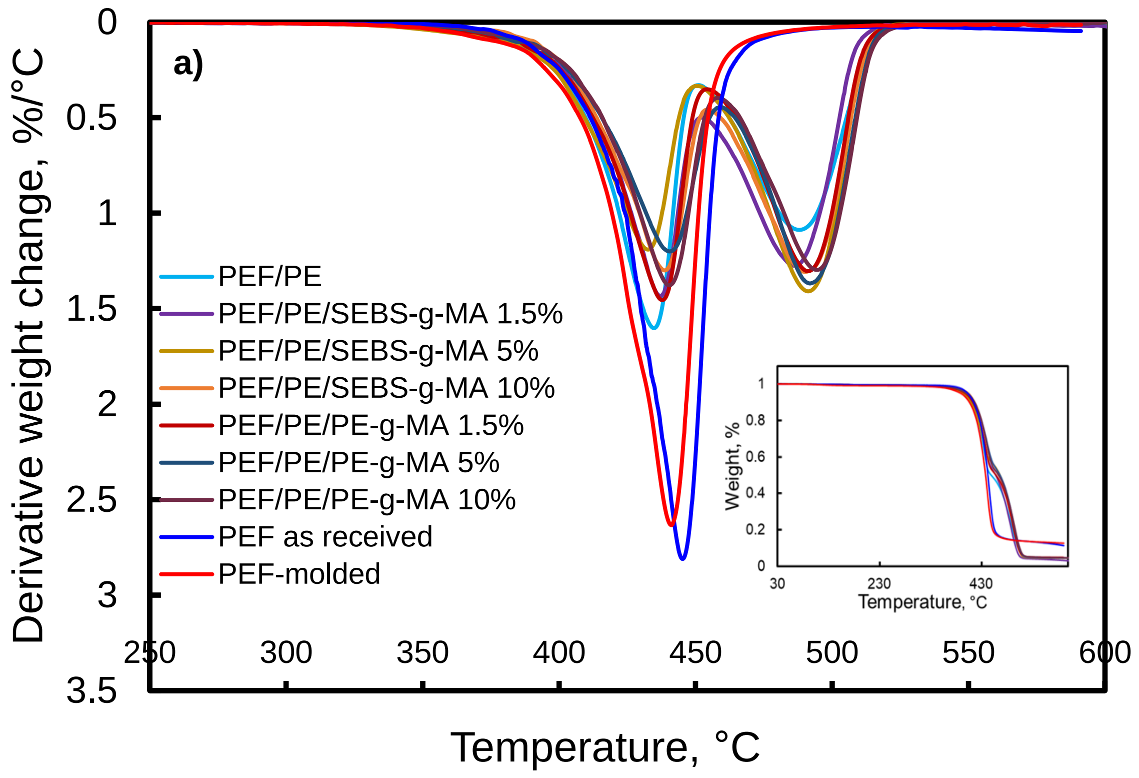

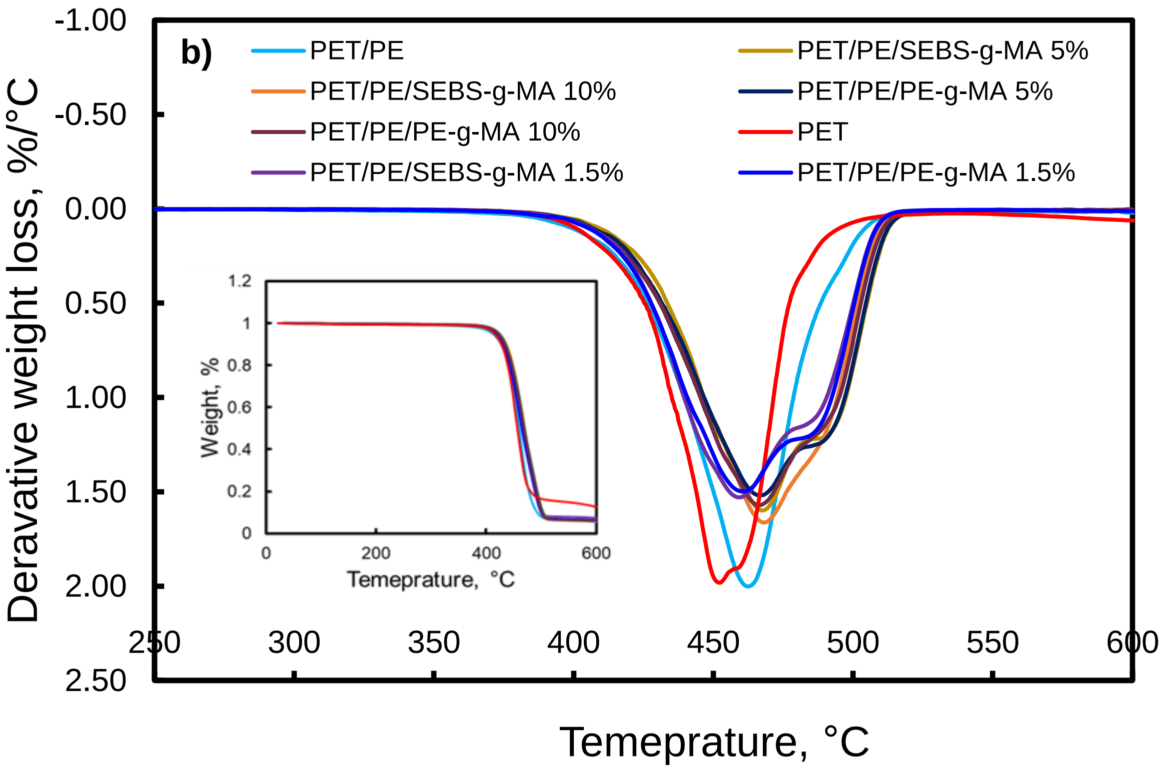

Figure S11 DGA, and TGA curves of a) PEF/PE blends, b) PET/PE blends

Table S9 Thermal stability of PEF/PE and PET/PE blends determined from TGA and DTG curves

**Sample**	**Temperature at 5% weight loss, °C**	**Temperature at 10% weight loss, °C**	**Temperature at max. derivative weight loss (Peak 1), °C**	**Temperature at max. derivative weight loss (Peak 2), °C**	**Residue at 640 °C, %**
PEF-As-received	402.8	414.4	446.0	-	0.93
PEF-molded	389.1	406.2	441.5.0	-	1.48
PEF/PE	394.3	410.1	435.0	489.0	4.52
PEF/PE/SEBS-g-MA	395.1	412.5	436.7	487.0	1.90
PEF/PE/SEBS-g-MA	392.6	408.8	433.0	491.0	4.35
PEF/PE/SEBS-g-MA	404.3	416.4	438.5	491.0	4.21
PEF/PE/PE-g-MA	397.5	412.5	438.0	491.0	4.88
PEF/PE/PE-g-MA	401.0	415.7	440.0	491.6	4.28
PEF/PE/PE-g-MA	402.8	416.8	440.0	494	4.49
PET-molded	414.9	427.5	453.0	-	9.9
PET/PE	411.8	426.8	462.5	-	4.37
PET/PE/SEBS-g-	421.5	432	460.5	485.0	6.8
PET/PE/SEBS-g-	425.1	437.5	468.0	491.0	6.02
PET/PE/SEBS-g-	422.0	434.2	468.0	488.0	5.36
PET/PE/PE-g-MA	419.5	431.2	461.0	484.0	5.9
PET/PE/PE-g-MA	423.3	434.6	468.0	491.0	5.77
PET/PE/PE-g-MA	421.5	433.4	467.0	488.0	5.63

DMA Test

We have analyzed cole–cole plots (E' vs E'' plot) for blends (compatibilized and uncompatibilized). Homogeneous polymeric systems are reported to show semi-circle diagram since two-phase systems show two modified semi-circles. The cole–cole plots of the blends are provided in Figure S12. showed imperfect semi-circles indicating heterogeneity of the systems.

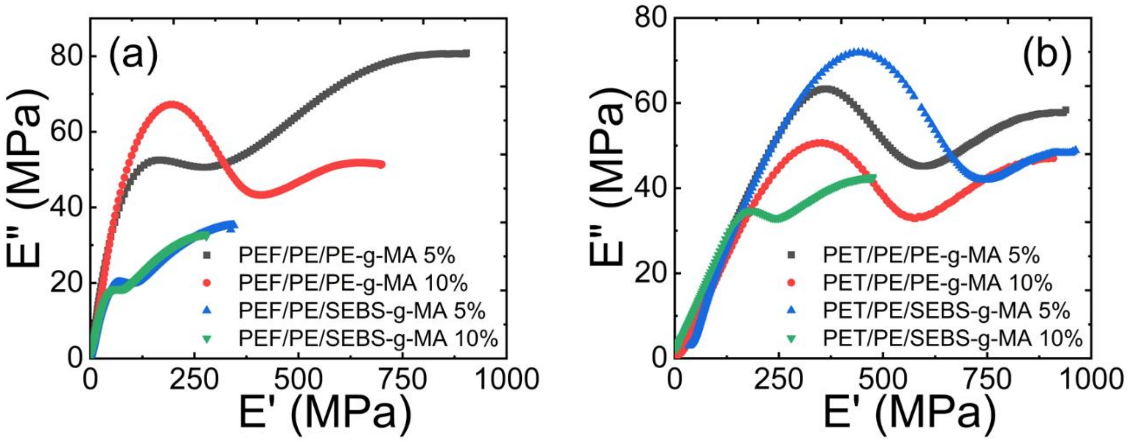

Figure S12 Cole-Cole plot of a) PEF/PE blends, b) PET/PE blends

Furthermore, though the concept of crosslinking density is conventionally used in chemically crosslinked polymers (thermosets or rubbers); herein, we have utilized this concept to understand chemical interactions between compatibilizers in the blends. The crosslink density was calculated according to the classical rubber elasticity theory proposed by Flory [25]:

$$\partial_c = \frac{E'}{3RT} \qquad (16)$$

Where ∂_c density (mol/m^3), R is the universal gas constant (8.314 J mol^{-1} K^{-1}), T is the thermodynamical temperature in the rubbery region (T_g + 30 °C) and E' is the storage modulus in the rubbery plateau. The values for crosslink density are listed in Table 4. It is noticed that crosslink density of all the PEF/PE compatibilized blends shows a slight increasing trend compared to pristine PEF and uncompatibilized PEF/PE indicating stronger interface due to chemical interactions. This behavior can be attributed to the degree of reaction between the compatibilizers and PEF, with PE-g-MA showing a more pronounced reaction with PEF as revealed by the complex viscosity data (in the supporting information). Nevertheless, study of the kinetics of compatibilization requires a complete separate investigation which is left for future targets from this study.

Table S10 Crosslinking Density in selected blends

	**T (°C)**	E' **(Pa)**	**Density (mol/m^3)**
**PEF**	105	2120	0.22
**PEF/PE**	123	1180	0.12
**PEF/PE/PE-g- MA 5%**	120	4120	0.42
**PEF/PE/PE-g- MA 10%**	120	5400	0.55

**PEF/PE/SEBS-g- MA 5%**	135	3540	0.35
**PEF/PE/SEBS-g- MA 10%**	123	3600	0.36

Mechanical Properties

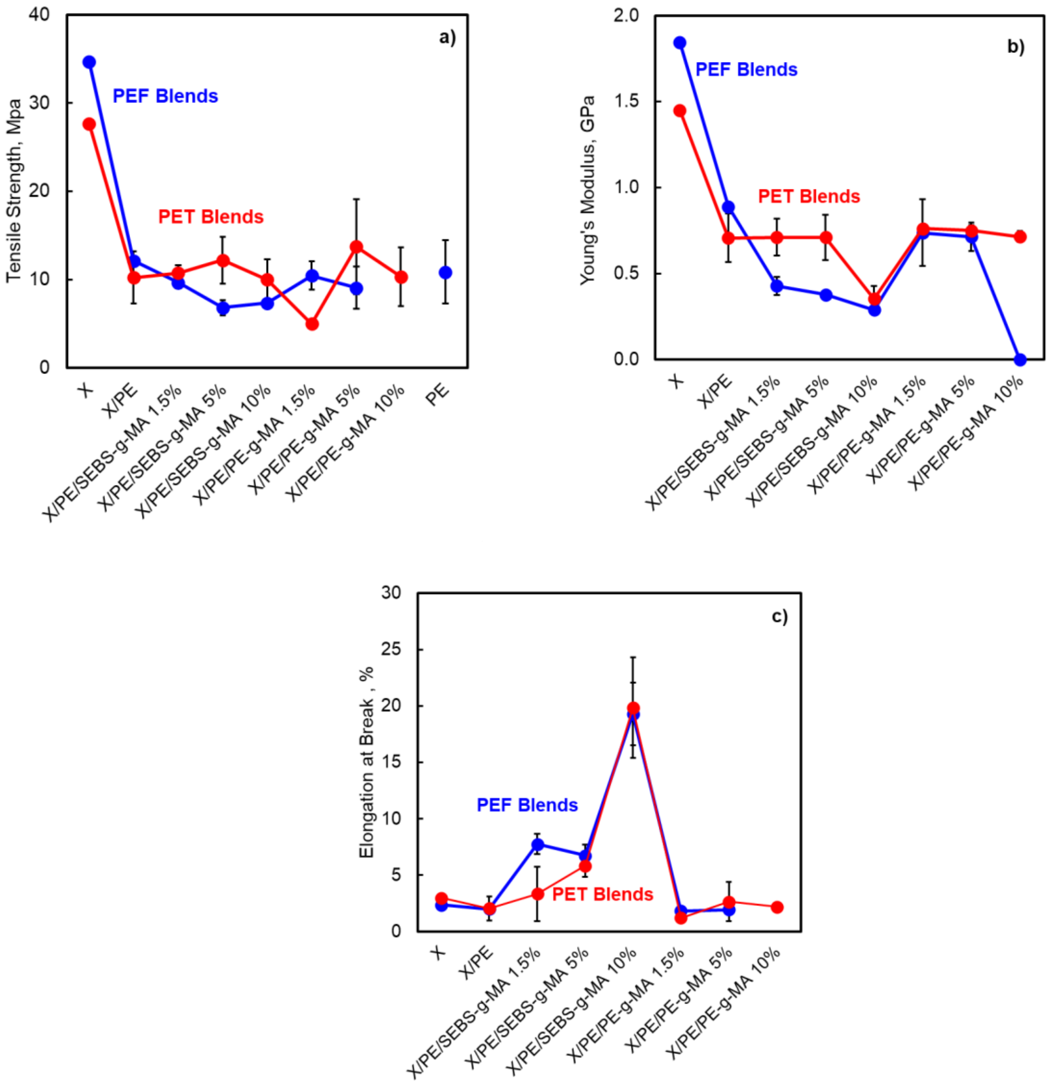

Figure S13 Mechanical Properties of PEF blends a) Tensile strength, b) Young's modulus, c) Elongation at break,

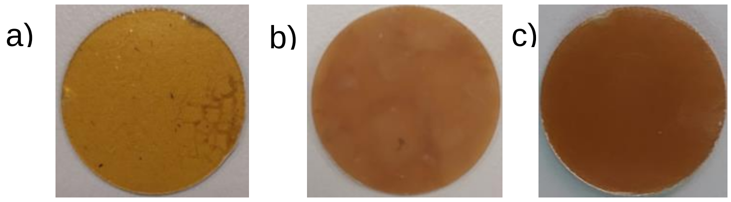

Figure S14 Disk samples of a) PEF, b)PEF/PE, c)PEF/PE/SEBS-g-MA 10%

References

1. Van Krevelen, D. W.; Te Nijenhuis K.; Cohesive Properties and Solubility. In Properties of Polymers, Their Correlation with Chemical Structure, Their Numerical Estimation and Prediction from Additive Group Contributions, 4th ed.; Van Krevelen, D. W., Te Nijenhuis K., Ed.; Elsevier: Amsterdam, 2009; pp 189-227.
2. Ravindra, R.; Krovvidi K. R.; Khan A. A. Solubility parameter of chitin and chitosan. *Carbohydr. Polym.* **1998**, *36*, 121-127.
3. Hoy, K. L.; The Hoy tables of solubility parameters. South Charleston, WV: Union Carbide Corp., Solvents & Coatings Materials, Research & Development Dept.; 1985.
4. Hoy, K. L. New values of the solubility parameters from vapor pressure data. *J. Paint Technol.* **1970**, *42*, 76–118.
5. Small, P. A. Some factors affecting the solubility of polymers. *J. Appl. Chem.* **1953**, *3*, 71-80.
6. Poulopoulou, N.; Smyrnioti D.; Nikolaidis G. N.; Tsitsimaka I.; Christodoulou E.; Bikiaris D. N.; Charitopoulou M. A.; Achilias D. S.; Kapnisti M.; Papageorgiou G. Z. Sustainable Plastics from Biomass: Blends of Polyesters Based on 2,5-Furandicarboxylic Acid. *Polymers.* **2020**, *12*, 225.
7. C. R, R.; Sundaran S. P.; A J.; Athiyanathil S. Fabrication of superhydrophobic polycaprolactone/beeswax electrospun membranes for high-efficiency oil/water separation. *RSC Advances.* **2017**, *7*, 2092-2102.
8. Kozbial, A.; Li Z.; Conaway C.; McGinley R.; Dhingra S.; Vahdat V.; Zhou F.; D'Urso B.; Liu H.; Li L. Study on the Surface Energy of Graphene by Contact Angle Measurements. *Langmuir.* **2014**, *30*, 8598-8606.
9. Van Krevelen, D. W.; Te Nijenhuis K.; Interfacial Energy Properties. In Properties of Polymers, Their Correlation with Chemical Structure, Their Numerical Estimation and Prediction from Additive Group Contributions, 4th ed.; Van Krevelen, D. W., Te Nijenhuis K., Ed.; Elsevier: Amsterdam, 2009; pp 227-241.
10. He, H.-Z.; Xue F.; Jia P.-F.; He G.-J.; Huang Z.-X.; Liu S.-M.; Xue B. Linear low-density polyethylene/poly(ethylene terephthalate) blends compatibilization prepared by an eccentric rotor extruder: A morphology, mechanical, thermal, and rheological study. *J. Appl. Polym. Sci.* **2018**, *135*, 46489-46498.
11. Vergnes, B. Average Shear Rates in the Screw Elements of a Corotating Twin-Screw Extruder. *Polymers.* **2021**, *13*, 304-315.
12. Jordhamo, G. M.; Manson J. A.; Sperling L. H. Phase continuity and inversion in polymer blends and simultaneous interpenetrating networks. *Polym. Eng. Sci.* **1986**, *26*, 517-524.
13. Miles, I. S.; Zurek A. Preparation, structure, and properties of two-phase co-continuous polymer blends. *Polym. Eng. Sci.* **1988**, *28*, 796-805.
14. Ho, R. M.; Wu C. H.; Su A. C. Morphology of plastic/rubber blends. *Polym. Eng. Sci.* **1990**, *30*, 511-518.
15. Utracki, L. A.; Shi Z. H. Development of polymer blend morphology during compounding in a twin-screw extruder. Part I: Droplet dispersion and coalescence—a review. *Polym. Eng. Sci.* **1992**, *32*, 1824-1833.
16. Stoclet, G.; Gobius du Sart G.; Yeniad B.; de Vos S.; Lefebvre J. M. Isothermal crystallization and structural characterization of poly(ethylene-2,5-furanoate). *Polymer.* **2015**, *72*, 165-176.
17. Oliveira, A. D. B.; Freitas D. M. G.; Araújo J. P.; Cavalcanti S. N.; Câmara D. S.; Agrawal P.; Mélo T. J. A. HDPE/LLDPE blends: rheological, thermal, and mechanical properties. *Mater. Res. Innovations.* **2020**, *24*, 289-294.

18. Torres-Huerta, A. M.; Palma-Ramírez D.; Domínguez-Crespo M. A.; Del Angel-López D.; de la Fuente D. Comparative assessment of miscibility and degradability on PET/PLA and PET/chitosan blends. *Eur. Polym. J.* **2014**, *61*, 285-299.
19. Araujo, C. F.; Nolasco M. M.; Ribeiro-Claro P. J. A.; Rudić S.; Silvestre A. J. D.; Vaz P. D.; Sousa A. F. Inside PEF: Chain Conformation and Dynamics in Crystalline and Amorphous Domains. *Macromolecules.* **2018**, *51*, 3515-3526.
20. Maini, L.; Gigli M.; Gazzano M.; Lotti N.; Bikiaris D. N.; Papageorgiou G. Z. Structural Investigation of Poly(ethylene furanoate) Polymorphs. *Polymers.* **2018**, *10*, 296-304.
21. Papageorgiou, G. Z.; Tsanaktsis V.; Bikiaris D. N. Synthesis of poly(ethylene furandicarboxylate) polyester using monomers derived from renewable resources: thermal behavior comparison with PET and PEN. *Phys. Chem. Chem. Phys.* **2014**, *16*, 7946-7958.
22. Chen, X.; Hou G.; Chen Y.; Yang K.; Dong Y.; Zhou H. Effect of molecular weight on crystallization, melting behavior and morphology of poly(trimethylene terephalate). *Polym. Test.* **2007**, *26*, 144-153.
23. Runt, J.; Rim P. B.; Howe S. E. Melting point elevation in compatible polymer blends. *Polym. Bull.* **1984**, *11*, 517-521.
24. Manias, E.; Utracki L. A.; Thermodynamics of Polymer Blends. In Polymer Blends Handbook; Utracki, L. A., Wilkie C. A., Ed.; Springer Netherlands: Dordrecht, 2014; pp 171-289.
25. Susan George, J.; Vijayan P P.; Ponçot M.; Keloth Paduvilan J.; Thomas S. Viscoelastic and rheokinetic behaviour of cellulose nanofiber/ cloisite 30B hybrid nanofiller reinforced epoxy nanocomposites. *Chem. Eng. J.* **2024**, *498*, 155170-155180.
